# Sterile Insect Technique in a *n*-patch System with Allee Effect and Mass Trapping: Modelling, Analysis and Simulations

**DOI:** 10.1007/s11538-026-01722-3

**Published:** 2026-09-23

**Authors:** Pierre-Alexandre Bliman, Manon de la Tousche, Yves Dumont

**Affiliations:** 1 Sorbonne Université, Université Paris Cité, CNRS, Inria, Laboratoire Jacques-Louis Lions, LJLL, EPC MUSCLEES, F-75005 Paris, France; 2 CIRAD, UMR AMAP, Pôle de Protection des Plantes, F-97410 Saint Pierre, France; 3 AMAP, Univ Montpellier, CIRAD, CNRS, INRAe, IRD, Montpellier, France; 4 Department of Mathematics and Applied Mathematics, University of Pretoria, Pretoria, South Africa

**Keywords:** Sterile insect technique, Mass trapping, Allee effect, Elimination, Metapopulation model, Monotone system theory, Optimal release strategy, Interior point algorithm, Oriental fruit fly, Numerical simulations.

## Abstract

The sterile insect technique (SIT) is a biological control method aimed at reducing or eliminating populations of pests and disease vectors. This technique involves releasing sterilised insects which, by mating with wild individuals, will reduce the target population. In this study, spatial aspects are incorporated through an explicit metapopulation model, in which both wild and sterile insects disperse across multiple patches. We derive a general sufficient condition ensuring elimination of the wild population in all patches through SIT. When a strong Allee effect is naturally present in each patch, the release programme can be terminated after a finite time, and an upper bound on the required release duration is provided. We then study an optimisation problem aimed at minimising the daily number of sterile insects released to ensure population elimination, under the constraint that the releases occur only in a prescribed subset of patches. For illustrative purposes, we explore through numerical simulations different dispersal network configurations. We also consider additional control by mass trapping (MT), which can affect the sterile insects entering trapped areas. The main contribution of this work is to enable the identification of the best spatial strategies for releasing sterile insects under practical constraints, in order to achieve population elimination.

## Introduction

Pests and disease vectors have become a critical global concern, impacting both food security and public health. In particular, fruit flies (Diptera: Tephritidae) cause substantial damage to vegetable crops and orchards. Among them, *Bactrocera dorsalis*, commonly known as the oriental fruit fly, is a major pest primarily found in tropical and subtropical regions (Baker et al. 2019) and is considered one of the world’s most invasive species (Clarke et al. 2005; Mutamiswa et al. 2021). Females lay eggs beneath the fruit’s surface, promoting bacterial and fungal growth that renders the fruit unmarketable. In African countries where *Bactrocera dorsalis* is established, high losses have been reported on mango crops. For example, in Mozambique, the percentage of damaged mango fruits ranged from 21% to 78% between September 2014 and August 2015 (Cugala et al. 2020). In Réunion Island, a French territory near Madagascar, mango infestation by fruit flies has greatly increased since the invasion of *Bactrocera dorsalis* in 2017 (Grechi et al. 2022).

To address these issues, a wide range of control methods have been developed, with varying degrees of success. Among the large variety of control techniques employed in the field, we focus on the Sterile Insect Technique (SIT). This biological control tool, developed by Knipling in 1955 (Knipling 1955), involves the mass production of the target insect, followed by the sterilisation of males (or both males and females, depending on the species) at the larval or pupal stage using ionising radiation. These sterilised insects are then released in large numbers into the field, where they mate with wild populations, leading to a gradual decline in the pest/vector population over time (Dyck et al. 2021).

Motivated by Integrated Pest Management and Integrated Vector Management strategies, we also consider additional treatments that increase mortality of the population. Mass trapping (MT) methods are widely used in the field and are specifically designed to target and increase adult mortality, through the combination of a semiochemical attractant, a killing agent—which is usually an insecticide (Barclay and Haniotakis 1991)—and a trapping device that is designed to attract and capture a large number of adult insects. While MT is useful for reducing wild populations, traps do not distinguish between wild and sterile individuals. As a result, they may also remove sterile insects from the system, which can reduce the overall efficiency of SIT if not properly accounted for. The success of the combined strategy thus depends both on the spatial deployment of control measures and on their potential interactions.

Since Knipling’s early work, a wide variety of SIT models—simple, sex-structured, and/or stage-structured—have been developed and extensively studied to improve the effectiveness of SIT, depending on the target pest or vector. Some examples include probabilistic models (Berryman 1967), computational models (Markhof 1973; Diouf et al. 2022), discrete models (Van Den Driessche 1985; Li and Yuan 2015; Barclay 2021), semi-discrete models (Huang et al. 2017; Strugarek et al. 2019; Aronna and Dumont 2020; Bliman et al. 2019; Dumont and Oliva 2024), and continuous models (Barclay and Mackauer 1980; Esteva and Yang 2005; Bliman et al. 2019; Aronna and Dumont 2020; Anguelov et al. 2012, 2020), with some incorporating tools from control theory (Thomé et al. 2010; Bliman et al. 2019; Almeida et al. 2022; Bliman and Dumont 2022; Bliman et al. 2024) to optimise release protocols or to design feedback control. However, except in Bliman and Dumont (2022), these models typically assume that the targeted area is isolated from surrounding areas–an idealised scenario that rarely holds in reality. In practice, the environment is heterogeneous, and both wild and sterile insects can disperse between neighbouring locations.

This spatial structure has important implications for release strategies. For example, the application of SIT often faces spatial limitations when certain areas are inaccessible for direct releases. Such constraints may arise from environmental conditions, such as dense forests or narrow mountainous terrain requiring aerial releases by helicopter for better manoeuvrability (Vargas et al. 1995), or from local stakeholders forbidding releases in their areas. A key advantage of SIT, however, is that sterile insects released in one location can disperse into neighbouring areas, potentially enabling suppression over a broader region even with localised releases.

Spatial dynamics of the SIT have been mainly studied using partial differential equations (PDEs). Early work, such as Lewis and Van Den Driessche (1993), examined wave extinction phenomena resulting from sterile insect releases, while later studies, including (Jiang et al. 2014), expanded on this framework. Other works, like (Dufourd and Dumont 2013), also used PDEs to include environmental factors, such as wind and vegetation patterns. However, the analysis of such models is challenging, especially in heterogeneous environments and when the spatial structure is complex.

An alternative to PDE models is the explicit metapopulation approach. In ecology, a metapopulation consists of a group of populations of the same species occupying distinct, homogeneous, and connected patches of suitable habitat within a larger landscape. Different modelling approaches have been developed to characterise metapopulation dynamics. In the classical, spatially implicit, approach introduced by Levins in 1969 (Levins 1969), local population dynamics are not described explicitly; instead, the model tracks the fraction of occupied patches, assuming that individuals have the same probability of moving to any habitat patch. By contrast, spatially explicit metapopulation models keep track of the evolution of the magnitude of the population in each patch and explicitly account for dispersal between patches, where movement typically depends on spatial proximity between habitat patches. This second approach is adopted in this work.

To our knowledge, all existing SIT metapopulation models (Bliman et al. 2024; Yang et al. 2020; Dumont et al. 2025) have been restricted to the two-patch case. In particular, a recent study (Dumont et al. 2025) established a sufficient condition for population elimination and subsequently optimised the release rates. This study also formulated and solved an optimal control problem aimed at reducing the population in a single patch below a prescribed threshold within finite time, while minimising the total number of sterile insects released.

By contrast, in the modelling of mass trapping (MT), the paper (Bliman et al. 2025) addressed the general case of *n* patches, deriving optimisation results for complete elimination that depend on the spatial network structure and the number of patches available for control.

The present paper develops a general *n*-patch model combining MT and SIT, in which both wild and sterile insects can disperse between neighbouring patches, with potentially distinct dispersal networks for wild and sterile populations. Unlike the two-patch case, the model accommodates arbitrary landscape configurations, including complex connectivity patterns and spatial heterogeneity that arise in realistic situations. In such a general framework, explicit analytical characterisations available in two-dimensional settings are no longer possible, making the development of new mathematical approaches necessary.

A general elimination condition is thus derived for this *n*-patch system. Based on this result, we provide a framework for determining where sterile insects should be released under practical constraints, such as a limited number of feasible release sites, in order to efficiently eliminate the wild population throughout the landscape.

Moreover, unlike most SIT models, the proposed framework can incorporate a natural strong Allee effect in each patch—which guarantees that the extinction equilibrium of the system without release is locally asymptotically stable—by adjusting the mating probability in each patch. This allows to study elimination in finite time and to analyse how long releases must be maintained. Specifically, once the system’s state enters the basin of attraction of the extinction equilibrium in the absence of release, the population will not recover even if the release efforts are subsequently relaxed. Below, we call release duration the time necessary to enter this basin.

The paper is organised as follows. In the next section, we introduce some notations and definitions that are relevant to our study. In Sect. 3, the uncontrolled model describing the natural evolution of the wild population is studied, first in a homogeneous isolated area, and then in a patchy environment. In particular, a numerically tractable condition that ensures population elimination is provided. Then in Sect. 4, we study the *n*-patch model describing the evolution of sterile insects with constant releases. In Sect. 5, the models for wild and sterile insects are coupled, and the feasibility of population elimination, under the constraint that the releases of sterile insects are limited to a prescribed subset of patches, is studied. When the (sufficient) condition for elimination feasibility is satisfied and when the origin of the uncontrolled system is locally asymptotically stable, we give an upper bound for the release duration. Then, in Sect. 6, an optimisation problem consisting of achieving elimination while minimising a certain cost function, chosen as a non-decreasing and convex function of the sterile insect release rates, is studied. Finally in Sect. 7, we use numerical simulations on *Bactrocera dorsalis* to study how different network configurations and practical constraints, including some where the releases are limited to a specific number of patches, influence release strategies. We also study SIT in combination with MT, and the numerical explorations suggest that, although MT increases the critical release rates required to eliminate the population, for fixed release rates it reduces the release duration, and thus decreases the total number of sterile insects released over the entire programme. For reader’s convenience, most of the proofs of the results are put in the Appendix.

## Notations and Definitions

Let us introduce some general notations and definitions that will be used in this paper.

### Definition 2.1

For any $z \in \mathbb {R}$, its *positive part* is defined as

$$ z^+:= \max \{0,z\}. $$

The inequalities between vectors are considered in their usual coordinate-wise sense, that is, for any $x = (x_1,\ldots ,x_n)$ and $y = (y_1,\ldots ,y_n)$,

- $$x \le y \Leftrightarrow x_i \le y_i, ~i = 1, \ldots , n$$,
- $$x < y \Leftrightarrow x \le y, ~ x \ne y$$,
- $$x \ll y \Leftrightarrow x_i < y_i, ~i = 1, \ldots , n$$.

A vector *x* is said to be *non-negative* if $x \ge 0_n$, *positive* if $x > 0_n$, and *strictly positive* if $x \gg 0_n$.

These definitions are extended to matrices.

Now, let $f: \mathbb {R}^n \rightarrow \mathbb {R}^n$. The function *f* is said:

- *non-decreasing* if $x \le y \Rightarrow f(x) \le f(y)$,
- *increasing* if x<y⇒f(x)<f(y),

Similarly, *f* is said *non-increasing* if -f is non-decreasing, and *decreasing* if -f is increasing.

### Definition 2.2

The extended positive real number line is denoted by

$$ \overline{\mathbb {R}}_+:= \mathbb {R}_+ \cup \{+ \infty \}. $$

Now, we recall the definition of a Metzler matrix.

### Definition 2.3

A matrix $A \in \mathbb {R}^{n \times n}$ is called *Metzler* if all its off-diagonal components are non-negative. Moreover, *A* is said to be *irreducible* if it is not similar via a permutation to a block triangular matrix; otherwise, *A* is said to be *reducible*.

### Definition 2.4

The *stability modulus* of a matrix $A \in \mathbb {R}^{n \times n}$ is defined as

$$ s(A):= \{\max \{{ \Re (\lambda )} \}: \lambda \text { is an eigenvalue of } A\}. $$

### Definition 2.5

A matrix $A \in \mathbb {R}^{n \times n}$ is said *Hurwitz* if s(A)<0.

We now introduce the following theorem. Its original form, known as the *Perron–Frobenius theorem* (see, for instance, (Berman and Plemmons, 1994, Theorem 1.4, Chapter 2)), is stated for non-negative matrices. Its extension to the class of Metzler matrices follows directly from the spectral shift property of eigenvalues.

### Theorem 2.1

(Extension of the Perron-Frobenius theorem) Let $A \in \mathbb {R}^{n \times n}$ be a Metzler matrix. Then, *A* has a positive right (resp. left) eigenvector. This right (resp. left) eigenvector is associated with a real eigenvalue, called the Perron-Frobenius eigenvalue, that is equal to *s*(*A*).

If moreover *A* is irreducible, then *s*(*A*) is a simple eigenvalue of *A*. Furthermore, *A* has a strictly positive right (resp. left) eigenvector associated with the Perron-Frobenius eigenvalue, and any positive right (resp. left) eigenvector of *A* is a scalar multiple of the previous one.

### Definition 2.6

The cardinal of any subset $\mathcal {C}$ of $\{1,\ldots ,n\}$ is denoted by $n_\mathcal {C}$. For any $x = (x_1,\ldots ,x_n)$, the point of $\mathbb {R}^{n_{\mathcal {C}}}$ composed of the $n_{\mathcal {C}}$ components of *x* with index in $\mathcal {C}$ is denoted by $x|_{\mathcal {C}}$:

$$ x|_{\mathcal {C}}:= \left( x_i \right) _{i \in \mathcal {C}}. $$

Similarly, for any matrix $A \in \mathbb {R}^{n \times n}$, define the submatrix

$$ A|_{\mathcal {C}}:= \left( A_{ij} \right) _{i,j \in \mathcal {C}}. $$

We now recall the definition of the Kronecker delta.

### Definition 2.7

For any $x,y \in \mathbb {R}$, define the Kronecker delta $\delta _x^y$ as

$$ \delta _x^y:= \left\{ \begin{array}{ll} 1 & \text{ if } x = y \\ 0 & \text{ otherwise. } \end{array} \right. $$

Last, we recall the definition of a cooperative and irreducible system of ODEs (Smith 1995).

### Definition 2.8

Let $\mathcal {D}$ be an open *p*-convex subset of $\mathbb {R}^n$, that is, $t x + (1-t) y \in \mathcal {D}$ for all t∈[0,1] whenever $x,y \in \mathcal {D}$ and x≤y. Consider the system of ODEs $\dot{x} = f(x)$, where $f: \mathcal {D}\rightarrow \mathbb {R}^n$ is differentiable. The system is *cooperative* on $\mathcal {D}$ if the Jacobian matrix of *f* is Metzler for all $x \in \mathcal {D}$. It is *irreducible* on $\mathcal {D}$ if the Jacobian matrix of *f* is irreducible for all $x \in \mathcal {D}$.

## Pest/Vector Population Model and Preliminary Results

In Sect. 3.1, the model describing the evolution of the wild population in a homogeneous isolated area is presented. Then in Sect. 3.2, we derive a general *n*-patch model describing the evolution of the population in presence of dispersal.

### One-Patch, Scalar Model

Let us introduce the following dynamical system:

1 $$\begin{aligned} \dot{x} = x \big ( p(x,a)b - \mu _1 -\rho - \mu _2 x\big ). \end{aligned}$$

The scalar *x*(*t*) represents the number of wild individuals capable of reproducing (adults) at time *t*, and $\dot{x}$ is the derivative of *x* with respect to time. The parameters b>0 and $\mu _1> 0$ represent the natural daily birth rate per individual and the natural daily death rate, respectively, while $\rho \in \mathbb {R}_+$ denotes the additional mortality rate induced by mass trapping. In fruit flies and mosquitoes, the immature phase, gathering eggs, larvae and pupae, is subject to competition for space and food resources (Burrack et al. 2009; Clarke 2019; Reinbold-Wasson and Reiskind 2021), as immature stages are unable to move to other sites when conditions become unfavourable. This intraspecific competition is taken into account by the term $-\mu _2 x^2$ in the model, with $\mu _2>0$.

For every $x,a \in \mathbb {R}_+$, the function

2 $$\begin{aligned} p(x,a):= \left\{ \begin{array}{ll} \dfrac{x}{x+a} & \text{ if } a > 0 \\ 1 & \text{ if } a=0 \end{array} \right. \end{aligned}$$

represents the density-dependent probability of a successful mating.

When a=0, this probability is equal to 1, and the growth is *logistic*. However, we know that in practice, depending on several environmental and/or biological factors, mating is not always guaranteed. More precisely, at low population densities, mating can become difficult, since males and females may have difficulties to find each other. To account for this effect, we consider a probability of mating, $\dfrac{x}{x+a}$, where a>0 is a parameter that models the difficulty for the species to mate (Durrett and Levin 1994; Dennis 1989). With this additional factor when a>0, we introduce what is called in ecology a *strong Allee effect* (Fauvergue 2013), meaning that at low density, the per capita growth rate of the population is negative, which implies that the extinction steady state is locally asymptotically stable. While an Allee effect is an issue for species that need to be protected (Stephens and Sutherland 1999), it is useful to eliminate pests and disease vectors. Another common approach to model a strong Allee effect is to introduce a factor $1-e^{-\beta x}$, where β is related to the mean distance an individual can effectively search for mates (McCarthy 1997). Both formulations lead to similar qualitative behaviour. We choose here the hyperbolic function $\frac{x}{x+a}$ because it has a direct interpretation as a mating probability and allows a unified treatment of both the low-density mating limitations and the impact of sterile insects. As will be seen later, the presence of sterile insects can be interpreted as an effective increase in the parameter *a*. In the remainder of this article, *a* is referred to as the *Allee parameter*.

When a=0, the mating conditions are perfect and the growth in model (1) is logistic. We introduce the *basic offspring number*

3 $$\begin{aligned} \mathcal {N}:= \frac{b}{\mu _1+\rho }, \end{aligned}$$

which represents the average number of offspring produced by an individual during its lifetime.

By denoting

$$ Q:= \frac{\mu _1+\rho }{\mu _2}, $$

one easily shows the following result.

#### Theorem 3.1

If a=0, then the following holds for (1):

1. If $\mathcal {N}\le 1$, then the origin 0 is the only non-negative equilibrium and is globally asymptotically stable (GAS) on $\mathbb {R}_+$.
2. If $\mathcal {N} > 1$, then there exists a unique positive equilibrium. Its value $x^*$ is given by
  $$ x^* = Q \left( \mathcal {N}-1 \right) . $$
  Moreover, this equilibrium is GAS on $\mathbb {R}^*_+$.

The following result is analogous to Theorem 3.1 in the case a>0. The equilibrium values were stated in Dumont et al. (2025); here, we also establish the basins of attraction and the stability properties of the equilibria, and a complete proof is provided in Appendix B.1, which was not included in Dumont et al. (2025).

#### Theorem 3.2

If a>0, then the origin 0 of (1) is locally asymptotically stable (LAS).

Moreover, let

$$ a^{\text {crit}}:= Q\left( \sqrt{\mathcal {N}}-1 \right) ^2. $$

The following holds for (1):

1. If $\mathcal {N} \le 1$, then the origin 0 is the only non-negative equilibrium and is GAS on $\mathbb {R}_+$.
2. If $$\mathcal {N} > 1$$,
  - If
    $$ a> a^{\text {crit}}, $$
    then the origin is the only non-negative equilibrium and is GAS on $\mathbb {R}_+$.
  - If
    $$ a= a^{\text {crit}}, $$
    then there exists only one positive equilibrium. Its value $x^*$ is given by
    $$ x^* =Q \left( \sqrt{\mathcal {N}}-1 \right) . $$
    Moreover, this equilibrium is unstable. The basins of attraction of 0 and $$x^*$$ in $$\mathbb {R}_+$$ are $$[0, x^*)$$ and $$[x^*,+\infty )$$, respectively.
  - If
    $$ 0< a< a^{\text {crit}}, $$
    then there exist exactly two positive equilibria $x_{\pm }$, with $x_- < x_+$. Their value is given by
    $$ x^*_{\pm } = \frac{Q}{2}\left( \mathcal {N}- 1 - \frac{a}{Q} \right) \left( 1 \pm \sqrt{1 - \frac{4a}{Q\left( \mathcal {N}-1- \frac{a}{Q} \right) ^2}}\right) . $$
    Moreover, $$x_-$$ is unstable and $$x_+$$ is stable. The basins of attraction of 0 and $$x_+$$ in $$\mathbb {R}_+$$ are $$[0, x_-)$$ and $$(x_-, +\infty )$$, respectively.

Now define the per capita growth rate

$$ g(x):= p(x,a)b - \mu _1 - \rho - \mu _2 x, $$

for *p*(*x*, *a*) defined in (2). By (Dumont et al., 2025, Sect. 3.2),

4 $$\begin{aligned} \max _{x \ge 0} g(x) = - \mu _1 - \rho + \left( \left( \sqrt{b} - \sqrt{a \mu _2} \right) ^{+}\right) ^2. \end{aligned}$$

We then establish the following result for population elimination. It is essentially a reformulation of the previous theorem, but with elimination now depending on the value of $\max _{x \ge 0} g(x)$. This result will serve as a basis for the generalisation to the case where the species evolves across interconnected areas, as discussed later in Sect. 3.2.

#### Theorem 3.3

The origin of (1) is GAS on $\mathbb {R}_+$ whenever

5 $$\begin{aligned} - \mu _1 - \rho + \left( \left( \sqrt{b} - \sqrt{a \mu _2} \right) ^+\right) ^2 < 0. \end{aligned}$$

Moreover, if $\mathcal {N}>1$, this condition is both necessary and sufficient for global asymptotic stability of the origin on $\mathbb {R}_+$.

#### Proof

See Appendix B.2 □

When the population evolves over *n* interconnected patches (see Sect. 3.2), an analogous stability condition will be derived, which generalises Theorem 3.3 (see Theorem 3.7 below).

### The *n*-Patch Pest/Vector Population Model

In Sect. 3.1, the model (1) typically assumes that the area in which the insects evolve is homogeneous and is isolated from surrounding areas–an idealised scenario that rarely holds in reality. In practice, the environment is heterogeneous, and insects can disperse between neighbouring locations. To incorporate this spatial structure, we now extend the model to a metapopulation framework consisting of *n* patches that are interconnected.

We first present the *n*-patch model in Sect. 3.2.1 and some local stability properties of the extinction equilibrium in Sect. 3.2.2. Then in Sect. 3.2.3, the existence of a maximal (i.e., componentwise largest) equilibrium is established, together with a stability property of this equilibrium. In Sect. 3.2.4, a sufficient condition for global elimination is provided. Finally in Sect. 3.2.5, we establish some monotonicity properties relative to the Allee parameter. These results will be applied later in Sect. 5, when incorporating the release of sterile insects into the model.

#### Wild Population Model

The *n*-patch metapopulation model obtained from the one-patch model (1) is the following system:

6 $$\begin{aligned} \dot{x}_i = x_i \big (p(x_i,a_i) b_i - \mu _{1,i} - \rho _i- \mu _{2,i} x_i\big ) + \sum \limits _{\begin{array}{c} j = 1\\ j \ne i \end{array}}^{n} d_{ij} x_j - \sum \limits _{\begin{array}{c} j = 1\\ j \ne i \end{array}}^{n} d_{ji} x_i, \qquad i = 1,\ldots ,n, \end{aligned}$$

which extends the logistic-type growth framework studied in Bliman et al. (2025) by incorporating an Allee effect in the patches. In the remaining of the present paper, system (6) is referred to as the *uncontrolled system*.

For every i=1,…,n, $x_i(t)$ is a scalar representing the number of individuals in the *i*-th patch at time *t* and $\dot{x}_i$ is its time derivative. The parameters $b_i, \mu _{1,i}$, $\mu _{2,i}$ are positive, $\rho _i \in \mathbb {R}_+$, and the function

7 $$\begin{aligned} g_i(x_i) := p(x_i,a_i)b_i - \mu _{1,i} - \rho _i- \mu _{2,i} x_i \end{aligned}$$

is the per capita growth rate of the population in the *i*-th patch, for *p* defined in (2). In particular, having $a_i>0$ corresponds to a strong Allee effect in Patch *i*, since as mentioned in Sect. 3.1, the local per capita growth rate $g_i$ becomes negative when the density in the patch is low. Note however that, contrary to the one-patch model (1), a strong Allee effect in a patch does not automatically imply the extinction of its population when its density is low, as immigration from other patches can potentially rescue it from collapsing.

Finally, $d_{ij} \in \mathbb {R}_+$ is a non-negative dispersal coefficient describing the flow of individuals from Patch *j* to Patch *i* (i≠j). Thus, define the connectivity matrix *D* of the wild population as follows:

$$ D_{ij}:= {\left\{ \begin{array}{ll} d_{ij} & \text { if } i \ne j \\ -\sum \limits _{\begin{array}{c} k=1 \\ k \ne i \end{array}}^{n} d_{ki}&\text { if } i=j. \end{array}\right. } $$

This matrix is Metzler, i.e., its non-diagonal components are non-negative. In some results of this paper, we will assume that *D* is irreducible, i.e., that the underlying directed graph is irreducible, implying that the species can migrate between any patches.

Denote $x:= (x_1,\ldots ,x_n)$, $a: = (a_1,\ldots ,a_n)$, and define $f_a(x)$ as the right-hand side of system (6), i.e.,

8 $$\begin{aligned} f_a(x) := \textrm{diag}\big ( p(x_i,a_i) b_i - \mu _{1,i} - \rho _i- \mu _{2,i} x_i \big ) x + Dx. \end{aligned}$$

When n=2, this type of system has been studied in the context of sterile insect releases in Dumont et al. (2025). This section generalises some results from that paper to an arbitrary number *n* of patches.

#### Local Stability Properties

This section establishes some local stability properties of the extinction equilibrium of (6). To this aim, denote by $J_a$ the Jacobian matrix at the origin of the function $f_a$ defined in (8). It writes

9 $$\begin{aligned} J_a = \textrm{diag}\left( b_i \delta _{a_i}^0 - \mu _{1,i} - \rho _i\right) + D, \end{aligned}$$

where $\delta ^0_{a_i}$ denotes the Kronecker delta.

##### Theorem 3.4

(Local stability of the extinction equilibrium) For any $a \in \mathbb {R}^n_+$, if $s(J_a) < 0$, then the origin of (6) is LAS.

In particular, if $a \gg 0_n$, then the origin is LAS.

##### Proof

When $s(J_a) < 0$, the origin of (6) being LAS is a classical result in stability theory.

When $a \gg 0_n$, one has $J_a = -\textrm{diag}(\mu _{1,i}+\rho _i) + D$. Since $\textbf{1}_n^T J_a = -\left( \mu _1+\rho _1,\ldots ,\mu _n+\rho _n \right)$, the Metzler matrix $J_a$ is strictly column diagonally dominant, and is therefore Hurwitz, implying the local asymptotic stability of the origin. □

##### Remark 3.1

From Theorem 3.4, a strong Allee effect in each patch ($a \gg 0_n$) implies the local asymptotic stability of the origin. However, the local asymptotic stability of the origin does not imply the presence of a strong Allee effect in every patch. Consider for example a two-patch model (n=2) where Patch 1 does not exhibit a (strong) Allee effect ($a_1=0$) while Patch 2 does ($a_2>0$). Assuming Patch 1 is intrinsically viable ($b_1 > \mu _{1,1} + \rho _1$), the origin can still become LAS if the dispersal rate from the viable patch to the patch with strong Allee effect satisfies:

$$\begin{aligned} d_{21} > (b_1-\mu _{1,1}-\rho _1)\left( 1+ \frac{d_{12}}{\mu _{1,2}+\rho _2} \right) . \end{aligned}$$

In this scenario, the extinction of the population when densities are low in both patches is not caused by a strong Allee effect in each patch, but rather by a dispersal-induced phenomenon where the emigration rate $d_{21}$ drains individuals from Patch 1 into Patch 2 faster than Patch 1’s intrinsic low-density growth rate can compensate, while Patch 2 acts as a sink due to its strong Allee effect.

##### Definition 3.1

Denote by $\mathcal {B}_a$ the basin of attraction of the origin of system (6).

In the next result, we give an approximation of $\mathcal {B}_a$.

##### Theorem 3.5

(Estimation of the basin of attraction) Let $a > 0_n$. Assume *D* is irreducible and $s(J_a) < 0$, and let $c \gg 0_n$ be a corresponding left (Perron) eigenvector of $J_a$, normalised so that $\min _i c_i = 1$. Then,

$$\begin{aligned} \left\{ x \in \mathbb {R}^n_+: \Psi \left( c^T x\right) < -s(J_a) \right\} \subset \mathcal {B}_a, \end{aligned}$$

where, for every $z \in \mathbb {R}_+$,

$$ \Psi (z):= \max _{\begin{array}{c} 1 \le k \le n \\ a_k> 0 \end{array}} \phi _k(z), \qquad \phi _k(z):= {\left\{ \begin{array}{ll} b_k \dfrac{z}{z + a_k} - \mu _{2,k} z & \text {if } z \le -a_k + \sqrt{\dfrac{a_k b_k}{\mu _{2,k}}} \\ \left( \left( \sqrt{b_k} - \sqrt{a_k \mu _{2,k}} \right) ^+\right) ^2 & \text {if } z > -a_k + \sqrt{\dfrac{a_k b_k}{\mu _{2,k}}} \end{array}\right. }. $$

Moreover, the previous set contains the origin.

##### Proof

See Appendix C. □

#### Maximal Equilibrium

Using the theory of cooperative systems, we establish the existence of a maximal equilibrium of system (6).

##### Theorem 3.6

(Properties of the maximal equilibrium) For any $a \in \mathbb {R}^n_+$, the system (6) admits a non-negative equilibrium $x_+(a)$ that is maximal, in the sense that any other equilibrium $x^*_a$ satisfies $x^*_a \le x_+(a)$. Moreover, $x_+(a)$ is GAS on the set $\{x \ge x_+(a) \}$.

Last, if *D* is irreducible, then any two non-negative equilibria $x^*_a$ and $\tilde{x}^*_a$ satisfy

10 $$\begin{aligned} x^*_a < \tilde{x}^*_a \Rightarrow x^*_a \ll \tilde{x}^*_a. \end{aligned}$$

##### Proof

See Appendix D. □

Notice that when *D* is irreducible and since the origin is an equilibrium, it follows from (10) that either the population is eliminated in all patches or it persists in all of them.

##### Remark 3.2

An immediate consequence of Theorem 3.6 is that if $x_+(a) = 0_{n}$, then it is GAS on $\mathbb {R}^n_+$.

##### Remark 3.3

The existence of a unique positive equilibrium for system (6) does not necessarily imply its global asymptotic stability on $\mathbb {R}^n_+ \setminus \{ 0_n\}$. Indeed, in such case and when the origin is LAS, then by monotonicity of system (6), the origin attracts every trajectory starting in the set $\{x: 0_n \le x < x_+(a) \}$ (Smith, 1995, Theorem 2.2, Chapter 2). This represents a significant difference compared to the case of perfect mating conditions ($a = 0_n$), for which the existence of a positive equilibrium implies both its uniqueness and its global asymptotic stability on $\mathbb {R}^n_+\setminus \{0_n\}$.

#### A Sufficient Condition for Elimination

In this section, we seek a condition guaranteeing that the extinction equilibrium of system (6) is GAS on $\mathbb {R}^n_+$, i.e., that the maximal equilibrium of (6) is null. To this aim, for all $a \in \mathbb {R}^n_+$, define the matrix

11 $$\begin{aligned} A(a) := \textrm{diag}\left( - \mu _{1,i} - \rho _i+ \left( \left( \sqrt{b_i} - \sqrt{a_i \mu _{2,i}} \right) ^+\right) ^2\right) + D. \end{aligned}$$

As noted in the discussion immediately preceding Theorem 3.3, each diagonal entry in $\textrm{diag}()$ corresponds to

12 $$\begin{aligned} \max _{x_i \ge 0} g_i(x_i), \end{aligned}$$

where $g_i$ is the per capita growth rate in Patch *i*, defined in (7). Thus, the matrix *A*(*a*) is the Jacobian matrix of system (6) evaluated at the unique point in the state space where the per capita growth rate is maximal in each patch.

Building on these notations, the following theorem is a direct application of (Lu and Takeuchi, 1993, Theorem 1).

##### Theorem 3.7

(Sufficient condition for global asymptotic stability of the origin) For any $a \in \mathbb {R}^n_+$, if s(A(a))<0, then $x_+(a) = 0_n$ and the origin of (6) is GAS on $\mathbb {R}^n_+$.

In the one-patch case (n=1), this theorem recovers the sufficient condition stated in Theorem 3.3. Notice that when $\mathcal {N} >1$, for $\mathcal {N}$ defined in (3), this condition is also necessary by Theorem 3.3.

In Theorem 3.7, only a sufficient condition for population elimination is established. When the mating conditions are perfect, i.e., when $a=0_n$, a stronger result can be obtained. In such case, the matrix $J_a$, defined in (9), satisfies

$$ J_a = J_0 = \textrm{diag}(b_i - \mu _{1,i}-\rho _i)+D, $$

and we have

$$ A(0) = J_0. $$

This case has been studied in Bliman et al. (2025). In particular, when *D* is irreducible, a necessary and sufficient condition for population elimination has been established in Theorem 2.2 of that paper, based on the sign of the stability modulus $s(J_0)$ of the matrix $J_0$. We extend this result to the case where *D* is not required to be irreducible.

##### Theorem 3.8

If $a=0_n$, then the following holds:

1. If $$s(J_0) \le 0$$, then $$x_+(0) = 0_n$$.
2. If $s(J_0) > 0$, then $x_+(0) > 0_n$. Moreover, $x_+(0) \gg 0_n$ if *D* is irreducible.

This theorem is a generalisation of the result given in Theorem 3.1 for the one-patch model (1). As a matter of fact, when n=1, the condition $s(J_0) \le 0$ is equivalent to $\mathcal {N} \le 1$.

##### Proof of Theorem 3.8

When *D* is irreducible, Theorem 3.8 is a direct application of (Bliman et al., 2025, Theorem 2.2). When *D* is not irreducible, one can decompose the underlying directed graph into its *strongly connected components* (*SCCs*), which form irreducible subgraphs (see Definition 4.4 introduced later in Section 4). Since *D* (and thus $J_0$) is reducible, there exists a permutation matrix such that $J_0$ can be brought into block triangular form where each block on the diagonal corresponds to an SCC, and thus is irreducible (see Varga (1999) p. 50). Therefore, if $s(J_0) \le 0$, then all the diagonal blocks have a non-positive stability modulus, and we deduce as a consequence of (Bliman et al., ( Bliman et al. (2025), Theorem 4.2) that $x_+(0) = 0_n$. Conversely if $s(J_0) > 0$, then there exists at least one diagonal block with a strictly positive stability modulus, and we deduce, still as a consequence of (Bliman et al., Bliman et al. (2025), Theorem 4.2), that $x_+(0) >0_n$. □

#### Monotonicity Properties

In this section, we establish some important monotonicity properties with respect to the Allee parameter *a*. These results will be applied to the SIT model in Sect. 5, since, as will be seen, the release of sterile insects artificially induces or amplifies a strong Allee effect in some patches, through an increase in the Allee parameter *a*.

##### Theorem 3.9

For any $a \in \mathbb {R}^n_+$, the maximal equilibrium $x_+(a)$ of system (6) is non-increasing with *a*, that is, for any $a,a'\in \mathbb {R}^n_+$,

13 $$\begin{aligned} a < a' \Rightarrow x_+(a') \le x_+(a). \end{aligned}$$

Moreover, if *D* is irreducible and $x_+(a) > 0_n$, then the inequality ≤ in (13) may be replaced by the inequality ≪.

Last, the basin of attraction $\mathcal {B}_a$ of the origin of system (6) is non-decreasing with *a*, that is, for any $a,a' \in \mathbb {R}^n_+$,

$$ a \le a' \Rightarrow \mathcal {B}_a \subset \mathcal {B}_{a'}. $$

##### Proof

See Appendix E.1. □

In particular, Theorem 3.9 states that when *D* is irreducible, then the increase of $a_i$ in *any* patch reduces the maximal equilibrium value in *every* patch of the network, which constitutes a strong result from a population reduction perspective.

From the inequality (13) in Theorem 3.9 and the fact that $x_+(a)$ is GAS on the set $\{x\ge x_+(a)\}$, one derives the following result.

##### Corollary 3.10

Let $$a \in \mathbb {R}^n_+$$. If the origin of (6) is GAS on $$\mathbb {R}^n_+$$, then the origin of system (6) with parameter $a^{\prime }$ is GAS on $$\mathbb {R}^n_+$$ for all $a^{\prime }\geq a$.

Finally, the following result provides monotonicty and convexity properties of the stability modulus of *A*(*a*). It will be useful later in Sect. 6 to study an optimisation problem related to the elimination of the wild population through the release of sterile insects.

##### Theorem 3.11

The function $a \in \mathbb {R}^n_+ \mapsto s(A(a))$ is convex and non-increasing in the following sense:

14 $$\begin{aligned} a < a' \Rightarrow s(A(a')) \le s(A(a)). \end{aligned}$$

Last, if *D* is irreducible, then this function is continuous on $\mathbb {R}^n_+$, and continuously differentiable and piecewise twice continuously differentiable on the set $\{ a \gg 0_n \}$. If in addition, $a_i < \frac{b_i}{\mu _{2,i}}$ for all i=1,…,n, then the inequality ≤ in (14) may be replaced by the inequality <.

##### Proof

See Appendix E.2. □

##### Remark 3.4

By Theorem 3.11, we have s(A(a))≤s(A(0)), for all $a\in \mathbb {R}^n_+$.

We now summarise the main results of this section. We have shown that a strong Allee effect in each patch ($a \gg 0_n$) implies the local asymptotic stability of the origin of (6) (Theorem 3.4) and an estimate of the basin of attraction of the extinction equilibrium is provided in Theorem 3.5. We have also proved the existence of a maximal equilibrium (Theorem 3.6). Then, in Theorem 3.7, we have derived a sufficient condition for this maximal equilibrium to be null, thereby guaranteeing elimination of the wild population in all patches. This condition depends on the stability modulus of the Jacobian matrix of system (6) evaluated at the unique point in the state space where the per capita growth rate is maximal in each patch. Finally, we have established monotonicity properties of the maximal equilibrium (Theorem 3.9) and of the stability modulus (Theorem 3.11) with respect to the Allee parameter *a*, in order to anticipate the effect of sterile insect releases, which is studied later in Sect. 5.

## The *n*-Patch Sterile Insect Model

We now consider the release of sterile insects. In Sect. 4.1, the model describing the evolution of the sterile population is presented, along with some relevant boundedness assumptions on the release rate. Then in Sect. 4.2, the asymptotic behaviour of the sterile insects is studied. Finally, in Sect. 4.3, the supremal sterile insect equilibrium for large admissible release rates is determined.

### Sterile Insect Model

We assume that sterile insects are released in each patch at a constant rate $\Lambda _i \in \mathbb {R}_+$ for i=1,…,n, with a mortality rate of $\mu _{s,i}>0$. The population of sterile individuals in patch *i*, denoted as $x_{s,i}$, is then modelled by:

15 $$\begin{aligned} \dot{x}_{s,i} = \Lambda _i - \left( \mu _{s,i} + \rho _{s,i}\right) x_{s,i} + \sum \limits _{\begin{array}{c} j = 1 \\ i \ne j \end{array}}^{n}d^s_{ij} x_{s,j} - \sum \limits _{\begin{array}{c} j = 1 \\ i \ne j \end{array}}^{n}d^s_{ji} x_{s,i}, \qquad i = 1,\ldots ,n. \end{aligned}$$

The scalar $\rho _{s,i} \in \mathbb {R}_+$ represents the additional mortality rate induced by mass trapping in Patch *i*. Indeed, in the field, traps targeting wild insects may also capture sterile insects. We do not impose $\rho _i = \rho _{s,i}$, as the relative susceptibility to trapping of sterile insects depends on the types of traps used. Since sterile insects are released as adults, there is no inter- or intraspecific competition for resources, such that there is no density-dependent mortality rate as in the wild population.

Similarly to the coefficients $d_{ij}$ introduced in Sect.3.2 to describe the dispersal of wild insects, $d^s_{ij}$ is a non-negative coefficient describing the flow of sterile insects from Patch *j* to Patch *i* (i≠j). We then introduce the matrix $D^s$, where

$$\begin{aligned} D^s_{ij} := {\left\{ \begin{array}{ll} d^s_{ij} & \text { if } i \ne j \\ -\sum \limits _{\begin{array}{c} k=1 \\ k \ne i \end{array}}^{n} d^s_{ki}&\text { if } i=j. \end{array}\right. } \end{aligned}$$

Note that system (15) writes in vector form as

$$ \dot{x}_s = \Lambda + J^s x_s, $$

where $x_s:= \left( x_{s,i} \right) _{i = 1,\ldots ,n}$, $\Lambda = \left( \Lambda _i \right) _{i = 1,\ldots ,n}$, two vectors, and

16 $$\begin{aligned} J^s := D^s - \textrm{diag}(\mu _s+\rho _s), \end{aligned}$$

with $$\mu _s = \left( \mu _{s,i} \right) _{i = 1,\ldots ,n}$$ and $$\rho _s = \left( \rho _{s,i} \right) _{i = 1,\ldots ,n}$$.

We consider that in the field, the release of sterile insects may be limited and restricted to a specific subset of plots, due, for example, to environmental constraints that prevent control implementation or to the reluctance of local stakeholders to allow certain forms of intervention. Accordingly, we introduce a boundedness assumption on the sterile insect release rate Λ in (15), recalling that the extended positive real line $\overline{\mathbb {R}}_+$ has been defined in Definition 2.2.

#### Definition 4.1

Let $\overline{\Lambda } \in \overline{\mathbb {R}}_+^n$. Define the sets $\mathcal {C}^s_B$, $\mathcal {C}^s_I$ and $\mathcal {C}^s$ as follows:

17a $$\begin{aligned} \mathcal {C}^s_B := \{ i \in \{1,\ldots ,n\}\ :\ 0< \overline{\Lambda }_i < + \infty \},\qquad \mathcal {C}^s_I := \{ i \in \{1,\ldots ,n\}\ :\ \overline{\Lambda }_i = + \infty \} \end{aligned}$$

and

17b $$\begin{aligned} \mathcal {C}^s := \mathcal {C}^s_B \cup \mathcal {C}^s_I. \end{aligned}$$

#### Definition 4.2

Let $\overline{\Lambda } \in \overline{\mathbb {R}}^n_+$. Any vector $\Lambda \in \mathbb {R}^n_+$ such that $\Lambda \le \overline{\Lambda }$ is called $\overline{\Lambda }$*-admissible*.

### Asymptotic Behaviour of the Sterile Insect Population

In this section, we study the asymptotic behaviour of the solutions of system (15). First, the fact that the matrix $J^s$ defined in (16) is Metzler and Hurwitz (it is strictly column diagonally dominant) implies that $J^s$ is invertible, with $-\left( J^s\right) ^{-1} > 0_{n \times n}$ (Horn and Johnson, 1994, Theorem 2.5.3). Thus, for any sterile insect initial condition $x_{s,0}\in \mathbb {R}^n_+$, the solution $x_s(t, x_{s,0})$ of (15) satisfies

18 $$\begin{aligned} x_s(t, x_{s,0}) = e^{J^s t}x_{s,0}+ \left( J^s\right) ^{-1} \left( e^{J^s t}-I \right) \Lambda . \end{aligned}$$

When $D^s$ is irreducible, we more precisely have $-\left( J^s\right) ^{-1} \gg 0_{n \times n}$ ((Bullo et al., 2018, Theorem 10.3)). These observations yield the following result.

#### Theorem 4.1

For any $\overline{\Lambda }$-admissible $\Lambda \in \mathbb {R}^n_+$ the system (15) admits a unique equilibrium $x^*_s(\Lambda ) = -\left( J^s\right) ^{-1} \Lambda \in \mathbb {R}^n_+$ which is GAS. In particular, it is increasing with $\Lambda \in \mathbb {R}^n_+$, and the following holds:

- If $\Lambda > 0_n$, then $x^*_s(\Lambda )$ is positive $(x^*_s(\Lambda ) > 0_n)$.
- If $D^s$ is irreducible and $\Lambda > 0_n$, then $x^*_s(\Lambda )$ is strictly positive $(x^*_s(\Lambda ) \gg 0_n)$.

In what follows, the value of $x^*_s(\Lambda )$ on the patches is characterised more precisely, depending on the connectivity matrix $D^s$ of sterile insects and the release vector Λ. When the network of the sterile insects is irreducible, the sterile insect equilibrium is strictly positive. When it is not irreducible, a detailed examination of the network is required. Introducing $\Gamma _s$ as the underlying graph of the matrix $D^s$, we revert to the irreducible case by decomposing $\Gamma _s$ into its *strongly connected components* (SCCs), which are irreducible subgraphs (see Definition 4.4). Such a diakoptic approach has been used to determine asymptotic stability of linear cooperative systems (Greulich et al. 2019) and the GAS equilibrium of system (6) when $a = 0_n$ (Bliman et al. 2025).

Let us introduce some adequate notations and definitions from graph theory.

#### Definition 4.3

For any subgraph or set of subgraphs $\mathcal {G}$ of $\Gamma _{s}$, the vertex set of $\mathcal {G}$ is noted $V_{\mathcal {G}}$, and the number of vertices in $V_{\mathcal {G}}$ is noted $n_{V_{\mathcal {G}}}$.

We now introduce the definition of a *strongly connected component* (Bang-Jensen and Gutin 2008).

#### Definition 4.4

A *strongly connected component (SCC)* of a graph $\mathcal {G}$ is a maximal subgraph of $\mathcal {G}$ which is irreducible, i.e., no additional arcs or vertices from $\mathcal {G}$ can be included in the subgraph without breaking its property of being irreducible.

Assume $\Gamma _s$ admits *N* SCCs, where $1 \le N \le n$. The case N=1 corresponds to the situation where $D^s$ is irreducible, and N=n corresponds to the case where $\Gamma _s$ is composed of *n* SCCs, each composed of a single patch (in other words, the system (15) consists of *n* independent subsystems). It is a classical result that one may arrange the SCCs in an *acyclic ordering*, that is, into a sequence $\mathcal {G}_1, \ldots , \mathcal {G}_N$, so that there is no arc (no direct path) from $\mathcal {G}_j$ to $\mathcal {G}_i$ unless possibly if j<i (Bang-Jensen and Gutin, 2008, p. 17).

#### Definition 4.5

For any SCC $\mathcal {G}$ of $\Gamma _s$, the set of its *in-neighbouring* (resp. *out-neighbouring*) *SCCs*, noted $\mathcal {G}^{-}$ (resp. $\mathcal {G}^{+}$), is the set of other SCCs from which there exists a direct path leading to $\mathcal {G}$ (resp. that are reachable from $\mathcal {G}$ via a direct path), that is, for any SCC $\mathcal {H} \ne \mathcal {G}$:

$$ \mathcal {H} \in \mathcal {G}^{-} \Leftrightarrow \text{ there } \text{ exists } i \in V_{\mathcal {G}} \text{ and } j \in V_{\mathcal {H}} \text{ such } \text{ that } d^s_{ij} >0. $$

$$ ( \text{ resp. } \mathcal {H} \in \mathcal {G}^{+} \Leftrightarrow \text{ there } \text{ exists } j \in V_{\mathcal {G}} \text{ and } i \in V_{\mathcal {H}} \text{ such } \text{ that } d^s_{ij} >0. ) $$

The *in-degree* of $\mathcal {G}$ is the number of vertices in $\mathcal {G}^-$. In particular, $\mathcal {G}$ is of in-degree zero if $\mathcal {G}^- = \emptyset$.

Notice that an SCC $\mathcal {H}$ cannot belong simultaneously to $\mathcal {G}^-$ and $\mathcal {G}^+$, and that $\mathcal {H} \in \mathcal {G}^-$ if and only if $\mathcal {G} \in \mathcal {H}^+$.

Let us introduce the concept of upstream (resp. downstream) subgraphs.

#### Definition 4.6

For any SCC $\mathcal {G}$ of $\Gamma _s$, the set of its *upstream* (resp. *downstream*) *SCCs*, noted $\mathcal {G}^{up}$ (resp. $\mathcal {G}^{down}$), is the set of other SCCs from which there exists a path leading to $\mathcal {G}$ (resp. that are reachable from $\mathcal {G}$ via a path).

Intuitively, sterile insects can only move from an upstream SCC to a downstream SCC, or remain within the same SCC.

With these considerations, one may now introduce the following theorem, which refines Theorem 4.1 by providing a more precise characterisation of the positivity of the equilibrium point, depending on the patches where sterile insects are released and the network connectivity.

#### Theorem 4.2

The equilibrium $x^*_s(\Lambda )$ satisfies, for any SCC $\mathcal {G}$ of $\Gamma _s$:

19 $$\begin{aligned} x^*_s(\Lambda )|_{V_{\mathcal {G}}} = - \left( J^s|_{V_{\mathcal {G}}}\right) ^{-1} \left( \Lambda |_{V_{\mathcal {G}}} + \left( \sum \limits _{\begin{array}{c} j \in V_{\mathcal {G}^-} \end{array}}d^s_{ij} x^*_{s,j}(\Lambda ) \right) _{i \in V_{\mathcal {G}}} \right) . \end{aligned}$$

In particular, for any $\overline{\Lambda }$-admissible $\Lambda \in \mathbb {R}^n_+$ such that $\Lambda _j > 0$ for every $j \in \mathcal {C}^s$,

20 $$\begin{aligned} x^*_s(\Lambda )|_{V_{\mathcal {G}}} = {\left\{ \begin{array}{ll} \gg 0_{n_{V_{\mathcal {G}}}} & \text {if } \{V_{\mathcal {G}} \cup V_{\mathcal {G}^{\text {up}}}\} \cap \mathcal {C}^s \ne \emptyset , \\ 0_{n_{V_{\mathcal {G}}}} & \text {otherwise}. \end{array}\right. } \end{aligned}$$

#### Proof

See Appendix F.1. □

Theorem 4.2 is a corollary of the second point in Theorem 4.1. Indeed, Theorem 4.2 can be applied to the particular case where $D^s$ is irreducible. In such case, $\Gamma _s$ is composed of a single SCC, which is $\Gamma _s$ itself.

#### Remark 4.1

As a consequence of Theorem 4.2 and its proof, sterile insects disperse (and persist) throughout the entire network if and only if they are released in each SCC with in-degree zero (see Definition 4.5 for the definition of an in-degree).

**Fig. 1:**
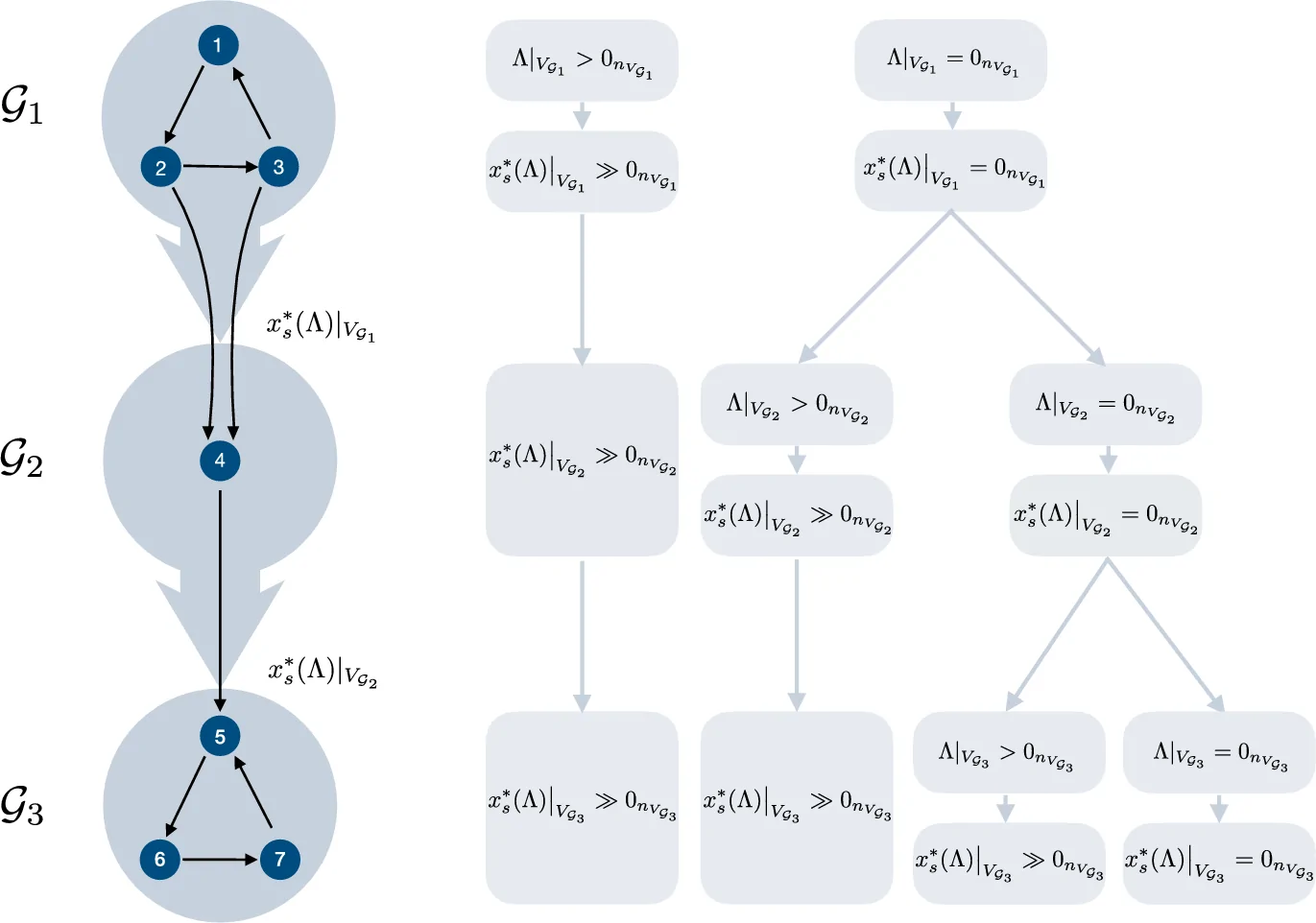
**Determination of the sterile insect equilibrium in the case of reducible graph.** The graph (left) is composed of three SCCs, namely $\mathcal {G}_1$, $\mathcal {G}_2$ and $\mathcal {G}_3$ in acyclic ordering. Upstream and downstream relations between SCCs are given by this ordering. The right figure depicts the various scenarios possible for the sterile insect equilibrium value

An example of reducible graph is illustrated in Fig. 1.

### Supremal Sterile Insect Equilibrium

We now evaluate the supremal sterile insect equilibrium $x^\infty _s$. For each admissible release vector Λ, we have shown in Theorem 4.1 that system (15) admits a unique equilibrium, which increases as Λ increases. The supremal sterile insect equilibrium is the upper bound of these equilibria over all $\overline{\Lambda }$-admissible release vectors Λ, and is obtained when each component of Λ is set to its maximal admissible value:

21 $$\begin{aligned} x^\infty _s := \sup \{x^*_s(\Lambda ): \Lambda ~ \overline{\Lambda }\text {-admissible} \} = \lim _{\Lambda \rightarrow \overline{\Lambda }} x^*_s(\Lambda ). \end{aligned}$$

The supremum is defined componentwise. The quantity $x^\infty _s$ will be useful for assessing the feasibility of wild insect elimination through SIT in Sect. 5, under constraints on the release rate Λ.

When $\mathcal {C}^s_I = \emptyset$, every component of the vector $\overline{\Lambda }$ is finite, which implies as a consequence of Theorem 4.1 that $x^\infty _s$ is simply equal to $- \left( J^s\right) ^{-1} \overline{\Lambda }$. When $\mathcal {C}^s_I \ne \emptyset$ and following the same approach as in Sect. 4.2, we now characterise $x_s^\infty$ depending on the connectivity matrix $D^s$. In what follows, we first consider in Sect. 4.3.1 the case where $D^s$ is irreducible. Building on this result, we then address in Sect. 4.3.2 the general case, without any assumption on the irreducibility of $D^s$.

#### Irreducible Sterile Insect Network

##### Lemma 4.3

Assume $\mathcal {C}^s_I \ne \emptyset$ and $D^s$ is irreducible. Then, the supremal sterile insect equilibrium $x^\infty _s$ satisfies

$$ x^\infty _{s,i} = + \infty , \quad i = 1,\ldots ,n. $$

##### Proof

By Theorem 4.1, for any $\overline{\Lambda }$-admissible Λ, one has

$$ x^*_s(\Lambda ) = - \left( J^s\right) ^{-1} \Lambda . $$

As seen before in Sect. 4.2, the matrix $\left( J^s\right) ^{-1}$ is strictly negative when $D^s$ is irreducible. Therefore, if $\Lambda _k \rightarrow +\infty$ for some $k \in \{1,\ldots ,n\}$, then $x^*_{s,i}(\Lambda ) \rightarrow \infty$ for all i=1,…,n. Since $\overline{\Lambda }_i = + \infty$ for any $i \in \mathcal {C}^s_I$, one has

$$ \lim _{\Lambda \rightarrow \overline{\Lambda }} x^*_{s,i}(\Lambda ) = +\infty , \quad i = 1,\ldots ,n. $$

This completes the proof of Lemma 4.3. □

#### Reducible Sterile Insect Network

##### Lemma 4.4

For any SCC $\mathcal {G}$ of $\Gamma _s$ and for any $k \in V_{\mathcal {G}}$, the supremal sterile insect equilibrium $x^\infty _s$ satisfies

$$ x^\infty _{s,k} = \left\{ \begin{array}{ll} +\infty & \text{ if } \{ V_{\mathcal {G}} \cup V_{\mathcal {G}^{\text {up}}} \} \cap \mathcal {C}^s_I \ne \emptyset \\ 0 & \text{ if } \{ V_{\mathcal {G}} \cup V_{\mathcal {G}^{\text {up}}} \} \cap \mathcal {C}^s = \emptyset \\ -\left( \left( J^s|_{V_{\mathcal {G}}}\right) ^{-1}\left[ \overline{\Lambda }|_{V_{\mathcal {G}}} + \left( \sum \limits _{\begin{array}{c} j \in V_{\mathcal {G}^-} \end{array}}{d^s_{ij} x^\infty _{s,j} } \right) _{i \in V_{\mathcal {G}}} \right] \right) _k \in (0, +\infty )&\text{ otherwise }. \end{array} \right. $$

##### Proof

See Appendix F.2. □

##### Remark 4.2

As a consequence of Lemma 4.4 and Remark 4.1, the sterile insect equilibrium can be made arbitrarily large in all patches if and only if the releases can be made arbitrarily large in each SCC with in-degree zero (see Definition 4.5 for the definition of an in-degree).

Let us summarise the main results of this section. We showed in Theorem 4.1 that the solutions of the sterile insect system (15) converge to a unique equilibrium. The result in Theorem 4.2 characterises in which patches the components of this equilibrium are positive, depending on the network structure and the release patches. Last, we also assessed in Lemma 4.4 how large this equilibrium can be under constraints on the release rates. These results will be useful to assess the feasibility of wild insect elimination through SIT in Sect. 5.

## General Properties of the SIT Model

The aim of this section is to adjust the release rate Λ in system (15) to drive the wild population towards elimination. This section is essentially an application of Sect. 3.2 since, as will be seen, the release of sterile insects artificially induces or amplifies a strong Allee effect in some patches, through an increase in the Allee parameter *a*.

In Sect. 5.1, the SIT model which consists in the coupling of the wild and sterile populations is presented. Next, in Sect. 5.2, we establish important properties of monotonicity with respect to the release rate. A sufficient condition for the feasibility of elimination of the wild population under some constraints on the release rates is then derived in Sect. 5.3. Finally, in Sect. 5.4, assuming this condition holds and that the origin of the uncontrolled system (6) is LAS, we provide an upper bound on the minimal release duration necessary to drive the wild population into the basin of attraction of the origin of the uncontrolled system (6).

### SIT Model

When sterile insects are released, they primarily disrupt the mating process. This implies that the birth rate of the wild population has to be modified to take into account the releases of sterile insects, as done in many other SIT models like (Bliman et al. 2019; Cai et al. 2014; Yang et al. 2020). Consequently, in each patch *i*, the term $\frac{x_i}{x_i + a_i}$ is replaced by $\frac{x_i}{x_i + a_i + \gamma x_{s,i}}$. This term represents the probability of a fertile mating occurring in Patch *i*, and γ is a parameter reflecting the average relative competitiveness of the sterile insects.

By coupling the wild population system (6) with the sterile insect system (15), we obtain the following SIT model of 2*n* ODEs:

22a $$\begin{aligned} \dot{x}_i&= x_i \big ( p(x_i, a_i + \gamma x_{s,i}) b_i - \mu _{1,i} - \rho _i -\mu _{2,i} x_i\big ) + \sum \limits _{\begin{array}{c} j = 1 \\ j \ne i \end{array}}^{n} d_{ij} x_j - \sum \limits _{\begin{array}{c} j = 1\\ j \ne i \end{array}}^{n} d_{ji} x_i, \end{aligned}$$

22b $$\begin{aligned} \dot{x}_{s,i}&= \Lambda _i - \left( \mu _{s,i} + \rho _{s,i}\right) x_{s,i}+ \sum \limits _{\begin{array}{c} j = 1 \\ i \ne j \end{array}}^{n}d^s_{ij} x_{s,j} - \sum \limits _{\begin{array}{c} j = 1 \\ i \ne j \end{array}}^{n}d^s_{ji} x_{s,i}, \qquad \qquad \qquad i = 1,\ldots ,n, \end{aligned}$$

where the function *p* has been defined in (2).

Using the formula in (18) for the solution $x(t,x_{s,0})$ of (22b) with sterile insect initial condition $x_{s,0}$, the wild insect population satisfies the *n*-dimensional system of time-varying ODEs:

23 $$\begin{aligned}&\dot{x}_i = x_i \Big ( p\big (x_i, a_i + \gamma x_{s,i}(t,x_{s,0})\big ) b_i - \mu _{1,i} - \rho _i -\mu _{2,i} x_i\Big ) + \sum \limits _{\begin{array}{c} j = 1\\ j \ne i \end{array}}^{n} d_{ij} x_j - \sum \limits _{\begin{array}{c} j = 1\\ j \ne i \end{array}}^{n} d_{ji} x_i, \nonumber \\&\mathrm{for~all}~i = 1,\ldots ,n. \end{aligned}$$

Since $x_s$ converges exponentially towards the equilibrium $x^*_s(\Lambda )$ by Theorem 4.1, one has by (Vidyasagar, 2003, Theorem 3.1) that the long-term behaviour of (23) is equivalent to that of the following *n*-dimensional autonomous system:

24 $$\begin{aligned}&\dot{x}_i = x_i \Big ( p\big (x_i, a_i + \gamma x^*_{s,i}(\Lambda )\big ) b_i - \mu _{1,i} - \rho _i -\mu _{2,i} x_i\Big ) + \sum \limits _{\begin{array}{c} j = 1\\ j \ne i \end{array}}^{n} d_{ij} x_j - \sum \limits _{\begin{array}{c} j = 1\\ j \ne i \end{array}}^{n} d_{ji} x_i, \nonumber \\&\mathrm{for~all}~i = 1,\ldots ,n. \end{aligned}$$

A central point here is that, by applying Theorem 3.6 with the substitution $a \mapsto a+ \gamma x^*_s(\Lambda )$, it follows that (24) admits a maximal equilibrium, noted $x_+(a+\gamma x^*_s(\Lambda ))$, which is GAS on the set $\{x \ge x_+(a+\gamma x^*_s(\Lambda ))\}.$

Theorem 3.7 also applies to system (24), yielding the following result, for the matrix function *A* defined in (11).

#### Theorem 5.1

If $s\big (A(a + \gamma x^*_s(\Lambda ))\big ) < 0$, then $x_+(a+\gamma x^*_s(\Lambda )) = 0_n$, and the origin of (24) is GAS on $\mathbb {R}^n_+$.

### Monotonicity Properties

We now introduce some monotonicity properties relative to the release rate Λ.

First, since $x^*_s(\Lambda )$ is a linear and increasing function of Λ by Theorem 4.1, the following result is derived from Theorem 3.9.

#### Theorem 5.2

The maximal equilibrium $x_+(a+\gamma x^*_s(\Lambda ))$ is a non-increasing function of Λ, that is, for any $\overline{\Lambda }$-admissible $\Lambda , \Lambda ' \in \mathbb {R}^n_+$,

25 $$\begin{aligned} \Lambda < \Lambda ' \Rightarrow x_+(a+\gamma x^*_s(\Lambda '))\le x_+(a+\gamma x^*_s(\Lambda )). \end{aligned}$$

Last, if *D* is irreducible and $x_+(a+\gamma x^*_s(\Lambda ))> 0_n$, the inequality ≤ in (25) may be replaced by the inequality ≪.

Theorem 5.2 implies that when *D* is irreducible, increasing the release rate in any single patch reduces the maximal equilibrium of the wild population in every patch of the network.

Similarly, the next result follows directly from corollary 3.10 and Theorem 3.11.

#### Theorem 5.3

If the origin of (24) is GAS on $$\mathbb {R}^n_+$$, then the origin of system (24) with release rate $\Lambda^{\prime }$ is GAS on $$\mathbb {R}^n_+$$ for all $$\Lambda ' \ge \Lambda $$.

Moreover, the function $\Lambda \in \mathbb {R}^n_+ \mapsto s\big (A(a + \gamma x^*_s(\Lambda ))\big )$ is convex and non-increasing in the following sense:

26 $$\begin{aligned} \Lambda < \Lambda ' \Rightarrow s\big (A(a + \gamma x^*_s(\Lambda '))\big ) \le s\big (A(a + \gamma x^*_s(\Lambda ))\big ) . \end{aligned}$$

Last, if *D* is irreducible, then this function is continuous on $\mathbb {R}^n_+$, and continuously differentiable and piecewise twice continuously differentiable on the set $\{\Lambda \in \mathbb {R}^n_+: a + \gamma x^*_s(\Lambda ) \gg 0_n \}$. If in addition $a_i + \gamma x^*_{s,i}(\Lambda ) < \frac{b_i}{\mu _{2,i}}$ for all i=1,…,n, the inequality ≤ in (26) may be replaced by the inequality <.

#### Proof

The first part of the proof is a direct application of corollary 3.10.

We only prove the convexity of the function $\Lambda \mapsto s\big (A(a + \gamma x^*_s(\Lambda ))\big )$, since the remainder of Theorem 5.3 follows by composing a↦s(A(a)) with the increasing function $\Lambda \mapsto a + \gamma x^*_s(\Lambda )$ and applying Theorem 3.11.

The function $\Lambda \mapsto a+ \gamma x^*_s(\Lambda )$ is affine. Since the function a↦s(A(a)) is convex by Theorem 3.11, we deduce by composition that the function $\Lambda \in \mathbb {R}^n_+ \mapsto s\big (A(a + \gamma x^*_s(\Lambda ))\big )$ is convex. □

### A Sufficient Condition for Wild Population Elimination Feasibility

In this section, we provide a sufficient condition under which, for given $\overline{\Lambda } \in \overline{\mathbb {R}}^n_+$, the elimination of the wild population may be achieved for some $\overline{\Lambda }$-admissible release rates $\Lambda \in \mathbb {R}^n_+$.

Since the stability modulus is a non-increasing function of the release rate (Theorem 5.3), our analysis focuses on evaluating the stability modulus of $A(a + \gamma x^*_s(\Lambda ))$ for large $\overline{\Lambda }$-admissible Λ. To this aim, denote

$$ A(a + \gamma x^\infty _s) = \lim _{\Lambda \rightarrow \overline{\Lambda }} A(a + \gamma x^*_s(\Lambda )), $$

where $x^\infty _s$ is the limit of $x^*_s(\Lambda )$ as $\Lambda \rightarrow \overline{\Lambda }$. Recall that an explicit formula for $x^\infty _s$ is provided in Lemma 4.4.

The quantity $A(a + \gamma x^\infty _s)$ is obtained from the matrix *A*(*a*) in (11), by modifying only the local diagonal terms. For indices *i* such that $x^\infty _{s,i} < + \infty$, the substitution $a_i \mapsto a_i + \gamma x^\infty _{s,i}$ is applied in the corresponding diagonal terms. For indices *i* such that $x^\infty _{s,i} = + \infty$, the diagonal local terms reduce to $-(\mu _{1,i}+\rho _i)$, reflecting the absence of local growth.

#### Lemma 5.4

For the matrix *A* defined in (11), one has

$$\begin{aligned} \inf \left\{ s\big (A(a + \gamma x^*_s(\Lambda ))\big ): \Lambda ~ \overline{\Lambda }\text{-admissible } \right\}&= s\big (A(a + \gamma x^\infty _s)\big ). \end{aligned}$$

#### Proof

Since $s\big (A(a + \gamma x^*_s(\Lambda ))\big )$ is a non-increasing function of $\overline{\Lambda }$-admissible Λ by Theorem 5.3, it follows that

$$ \inf \left\{ s\big (A(a + \gamma x^*_s(\Lambda ))\big ): \Lambda ~ \overline{\Lambda }\text{-admissible } \right\} = \lim _{\Lambda \rightarrow \overline{\Lambda }} s\big (A(a + \gamma x^*_s(\Lambda ))\big ). $$

By (21), one has that $\lim _{\Lambda \rightarrow \overline{\Lambda }} x^*_s(\Lambda ) = x^\infty _s$, which implies

$$ \lim _{\Lambda \rightarrow \overline{\Lambda }} A(a+\gamma x^*_s(\Lambda )) = A(a + \gamma x^\infty _s). $$

The equality in Lemma 5.4 then follows from the continuity of the stability modulus of a Metzler matrix. □

Building on Lemma 5.4, we derive a sufficient condition for the feasibility of population elimination. In addition, recall that $s(J_a) < 0$, for $J_a$ defined in (9), implies the local asymptotic stability of the origin of the uncontrolled model (6) (see Theorem 3.4). Thus, we also introduce the time required for a trajectory of the model with prescribed releases (23) to enter $\mathcal {B}_a$, the basin of attraction of the origin of the uncontrolled system (6).

#### Definition 5.1

For any wild and sterile insect initial conditions $x_0, x_{s,0}\in \mathbb {R}^n_+$, let $\tau (x_0,x_{s,0})$ denote the *release duration*, that is,

$$ \tau (x_0,x_{s,0}):= \inf \{t \ge 0: x(t,x_0, x_{s,0}) \in \mathcal {B}_a \}, $$

where $x(t,x_0,x_{s,0})$ is the solution of the system with prescribed releases (23), with wild and sterile insect initial conditions respectively equal to $x_0$ and $x_{s,0}$, and $\mathcal {B}_a$ is the basin of attraction of the uncontrolled system (6).

In addition, denote by $\Sigma _s(x_0, x_{s,0})$ the *total number of sterile insects released until entering the uncontrolled basin of attraction of the origin*, that is, for prescribed release rate vector Λ,

$$ \Sigma _s(x_0, x_{s,0}):= \textbf{1}^{\textsf {T}}_n \Lambda ~ \tau (x_0,x_{s,0}). $$

Departing from $x_0$, stopping the release of sterile insects for $t\ge \tau (x_0,x_{s,0})$ slows down the elimination, but does not suppress it.

The next result provides a sufficient condition for the feasibility of the elimination of the wild insect population within a finite time.

#### Theorem 5.5

If $s\big (A(a + \gamma x^\infty _s)\big ) < 0$, then there exists $\Lambda ^*$ $\overline{\Lambda }$-admissible such that $x_+(a+\gamma x^*_s(\Lambda ))= 0_n$ for any $\overline{\Lambda }$-admissible $\Lambda \ge \Lambda ^*$.

Moreover if $s(J_a) < 0$, then $\tau (x_0,x_{s,0}) < + \infty$ and $\Sigma _s(x_0,x_{s,0}) < + \infty$ for any $\Lambda \ge \Lambda ^*$ and any $x_0,x_{s,0}\in \mathbb {R}^n_+$.

#### Proof

If $s\big (A(a + \gamma x^\infty _s)\big ) < 0$, then by continuity of the stability modulus, there exists a $\overline{\Lambda }$-admissible $\Lambda ^*$ such that $s\big (A(a + \gamma x^*_s(\Lambda ))\big ) < 0$ for any $\overline{\Lambda }$-admissible $\Lambda \ge \Lambda ^*$. By applying Theorem 5.1, the origin of (24) is then GAS for for any $\Lambda \ge \Lambda ^*$, and $x_+(a+\gamma x^*_s(\Lambda ))= 0_n$.

If moreover $s(J_a)<0$, then the origin of the uncontrolled system (6) is LAS by Theorem 3.4. Since $x_+(a+\gamma x^*_s(\Lambda ))= 0_n$, there exists a time *t* such that $x(t,x_0,x_{s,0})$ enters the basin of attraction $\mathcal {B}_a$ of the uncontrolled system (6), implying $\tau (x_0,x_{s,0}) < + \infty$, and thus $\Sigma _s(x_0, x_{s,0}) < + \infty$. □

Theorem 5.5 states in particular that if elimination is feasible and $s(J_a)<0$, the release duration $\tau (x_0,x_{s,0})$ is finite: the releases can be stopped as soon as the solution of (24) enters the basin of attraction of the origin of the uncontrolled system (6). In contrast, as a consequence of Theorem 3.8, when $a = 0_n$ and $s(J_a)>0$, that is, the extinction equilibrium of (6) is unstable, the release must be maintained indefinitely to reach elimination in all patches, causing the total number $\Sigma _s(x_0,x_{s,0})$ of sterile insects released to increase linearly with time. However, notice that using non constant release values, e.g., values elaborated via a feedback law based on measurement of the wild population, may lead to stabilisation with a finite total release effort (see, e.g., Bhaya and Bliman (2025)).

Moreover, notice that when $\mathcal {C}^s_I \ne \emptyset$ and $D^s$ is irreducible, elimination is always feasible. Indeed in such case, we have by Lemma 4.3 that all components of $x^\infty _s$ are infinite. This implies that

$$ A(a + \gamma x^\infty _s) = -\textrm{diag}(\mu _{1,i}+\rho _i) + D. $$

This matrix is Metzler and strictly column diagonally dominant, and is therefore Hurwitz.

### Estimate of the Release Duration

In the previous section, we showed that when $s(J_a) < 0$ and $s(A( a + \gamma x^\infty _s) ) < 0$, elimination of wild insects may be achieved in finite time by large enough $\overline{\Lambda }$-admissible release rates Λ. In the following, building on the estimation of the basin of attraction of the origin of the uncontrolled system (6) in Theorem 3.5, we give an upper bound for the release duration $\tau (x_0,x_{s,0})$.

#### Theorem 5.6

Let $a > 0_n$. Assume *D* is irreducible and that $s(J_a) < 0$, and let $c \gg 0_n$ be a corresponding left (Perron) eigenvector of $J_a$, normalised so that $\min _i c_i = 1$.

Also assume that $s\big (A(a + \gamma x^\infty _s)\big )< 0$, and let Λ $\overline{\Lambda }$-admissible such that $\Lambda \ge \Lambda ^*$, for $\Lambda ^*$ defined in Theorem 5.5. Then, for any $x_0, x_{s,0}\in \mathbb {R}^n_+$,

$$\begin{aligned} \tau (x_0, x_{s,0}) \le \inf \left\{ t \ge 0 : \Psi \left( c^T x(t,x_0,x_{s,0})\right)< -s(J_a) \right\} < + \infty , \end{aligned}$$

where $x(t,x_0,x_{s,0})$ is the solution of the system with prescribed releases (23), and the function Ψ has been defined in Theorem 3.5.

In particular, when $x_{s,0}= 0_n$,

27 $$\begin{aligned} \tau (x_0, 0_n) \le \inf \left\{ t \ge 0 : \Psi \left( c^T x(t,x_0,0_n)\right)< -s(J_a) \right\} < + \infty . \end{aligned}$$

To apply this result, one must simulate the non-autonomous differential equation (23), and compute the evolution of the scalar $\Psi (c^{\textsf {T}} x(t))$ along the resulting trajectory.

#### Proof of Theorem 5.6

We first recall the result of Theorem 3.5:

$$\begin{aligned} \{ x \in \mathbb {R}^n_+ : \Psi ( c^{\textsf {T}} x) < -s(J_a) \} \subset \mathcal {B}_a, \end{aligned}$$

where $\mathcal {B}_a$ is the basin of attraction in $\mathbb {R}^n_+$ of the origin of the uncontrolled system (6). Therefore, one has

$$ \tau (x_0,x_{s,0}) \le \inf \left\{ t \ge 0: \Psi \left( c^T x(t,x_0,x_{s,0})\right) < -s(J_a) \right\} . $$

Finally, we prove that

28 $$\begin{aligned} \inf \left\{ t \ge 0 : \Psi \left( c^T x(t,x_0,x_{s,0})\right)< -s(J_a) \right\} < + \infty . \end{aligned}$$

As $\Lambda \ge \Lambda ^*$ for $\Lambda ^*$ defined in Theorem 5.5, one has thanks to that theorem that

$$ \lim _{t \rightarrow +\infty }c^T x(t,x_0,x_{s,0}) = 0. $$

Since $-s(J_a) > 0$ and Ψ(0)=0, we deduce by continuity of Ψ that (28) holds. This concludes the proof of the statement. □

Notice that, by cooperativeness of system (23), and since the solution $x_s(t,x_{s,0})$ of (22b) satisfies $x_s(t,x_{s,0}) \ge x_s(t,0_n)$, the upper bound provided in (27) when the sterile insect initial condition $x_{s,0}$ is null is valid for all $x_{s,0} \in \mathbb {R}^n_+$.

Moreover, still by monotonicity of the system (23), for all viable states, that is, for all wild insect initial condition $x_0 \in \{x: 0_n \le x <x_+(a) \}$, where $x_+(a)$ is the maximal equilibrium of the uncontrolled system (6), one has in particular

$$ \tau (x_0,x_{s,0}) \le \inf \left\{ t \ge 0: \Psi \left( c^T x(t,x_+(a),0_n)\right)< -s(J_a) \right\} < + \infty . $$

Also notice that the maximal equilibrium $x_+(a)$ can be easily computed numerically. When $a=0_n$, by Theorem 3.8, the maximal equilibrium $x_+(0)$ is GAS on $\mathbb {R}^n_+\setminus \{0_n\}$. Thus, for any initial condition $x_0>0_n$ the solution of (6) with $a=0_n$ converges to $x_+(0)$. For $a>0_n$, Theorem 3.6 shows that $x_+(a)$ is GAS on the set $\{x\ge x_+(a)\}$. Moreover, (13) implies

$$ x_+(a)\le x_+(0)\quad \text {for all }a>0_n. $$

Therefore, initialising the numerical solution of (6) at $x_+(0)$ yields convergence to $x_+(a)$.

We now summarise the main results of this section. We derived a sufficient condition for the elimination of the wild population through SIT in Theorem 5.1, and established some monotonicity properties with respect to the release rate in Theorem 5.2 and Theorem 5.3. This led to a sufficient condition for the feasibility of wild population elimination under constraints on the release rates in Theorem 5.5. Last, when the origin of the uncontrolled system (6) is LAS, we provided a finite upper bound for the release duration $\tau (x_0,x_{s,0})$ in Theorem 5.6.

## A Cost-Effective Approach for Achievable Elimination Scenarios

In this section, it is assumed that $s\big (A(a + \gamma x^\infty _s)\big )<0$, so that the wild population can be driven to elimination for sufficiently large $\overline{\Lambda }$-admissible release rates (see Theorem 5.5). We build a cost-efficient strategy to achieve elimination, while minimising a certain control cost. Here, this cost is taken as a weighted sum of the sterile insect release rates $\Lambda _i$, but the results presented below apply equally to the minimisation of any other convex, increasing and coercive function of Λ. Notice that a related optimisation problem consisting of optimising over additional mortality rates has been studied in Bliman et al. (2025).

We first introduce the minimisation problem and its solvability properties (Theorem 6.1) and address a monotonicity property of the solutions with respect to the Allee parameter *a* (Theorem 6.2). Then, in order to solve numerically the problem, we provide in Lemma 6.3 a formula for the gradient of the problem’s constraint, which relies on an explicit expression for the gradient of the stability modulus of an irreducible Metzler matrix with respect to its diagonal elements.

Let us introduce a fixed vector π, where for each $i \in \mathcal {C}^s$, $\pi _i > 0$ denotes the relative intervention price for the release of sterile insects in Patch i.

For any α such that $0< \alpha < - s\big (A(a + \gamma x^\infty _s)\big )$, let us introduce the following minimisation problem:

29 $$\begin{aligned} \begin{array}{ll} \text {minimise} & \pi ^{\textsf {T}} \Lambda \\ \text {subject to} & \Lambda ~\overline{\Lambda }\text {-admissible} \\ & h(\Lambda ) \le -\alpha , \end{array} \end{aligned}$$

where the constraint in (29) is defined by the function:

30 $$\begin{aligned} h(\Lambda ) := s\big (A(a + \gamma x^*_s(\Lambda ))\big ) . \end{aligned}$$

The quantity $\pi ^{\textsf {T}} \Lambda$ represents the total daily cost corresponding to the $\overline{\Lambda }$-admissible release rate $\Lambda \in \mathbb {R}^n_+$. Since α>0, the constraint $h(\Lambda ) \le - \alpha$ ensures global asymptotic stability of the origin of (24) (see Theorem 5.1).

The solvability of problem (29) is addressed in the next result. The proof is exactly the same as for Theorem 5.1 in Bliman et al. (2025) and thus is not provided here. It relies on the monotonicity and convexity properties of the function *h* (Theorem 5.3).

### Theorem 6.1

Let α satisfy $s\big (A(a + \gamma x^\infty _s)\big )< -\alpha < 0$. Then Problem (29) admits local minimisers. The latter are also global and their set is convex. Moreover, the following holds:

1. If s(A(a))>-α, then any minimiser $\Lambda ^*$ satisfies $\Lambda ^* > 0_n$ and $h(\Lambda ^*)=-\alpha$.
2. If $s(A(a)) \le -\alpha$, then the minimiser is unique and equal to $0_n$.

### Theorem 6.2

Let $a,a' \in \mathbb {R}^n_+$ and let α satisfy $s\big (A(a + \gamma x^\infty _s)\big )< -\alpha < 0$. Let $\Lambda _{a'}^*, \Lambda _a^*$ be corresponding minimisers of Problem (29). Then,

$$ a \le a' \Rightarrow \pi ^{\textsf {T}} \Lambda ^*_{a'} \le \pi ^{\textsf {T}} \Lambda ^*_{a}. $$

### Proof

For any $a^{\prime }\geq a$, we have by Theorem 3.11 that $$s\big (A(a' + \gamma x^\infty _s)\big ) \le s\big (A(a + \gamma x^\infty _s)\big ) <0$$. Hence, by Theorem 6.1, Problem (29) with parameters *a* and $a^{\prime }$ admits global minimisers.

Let $$\Lambda ^*_a$$ and $$\Lambda ^*_{a'}$$ be minimisers of Problem (29) with parameters *a* and $a^{\prime }$, respectively. Since $a^{\prime }\geq a$, one has, still by Theorem 3.11, that

$$ s\big (A(a' + \gamma x^*_s(\Lambda ^*_a))\big ) \le s( A(a + \gamma x^*_s(\Lambda ^*_a)))= -\alpha , $$

where the last equality is a consequence of Theorem 6.1. Therefore, $$\Lambda ^*_a$$ satisfies the constraints of Problem (29) with parameter $a^{\prime }$. By definition of a minimiser, one deduces that $$ \pi ^{\textsf {T}} \Lambda ^*_{a'} \le \pi ^{\textsf {T}} \Lambda ^*_a.$$ □

By Theorem 6.1, (29) is a convex minimisation problem. It can be solved efficiently by an interior-point method (Boyd and Vandenberghe, 2004, Chapter 11), slightly adapted from Bliman et al. (2025), where the optimisation was performed with respect to the additional mortality rates $\rho _i$. Interior-point algorithms are particularly well-suited for convex problems with constraints, as they approach the optimal solution from the interior of the feasible region while ensuring constraint satisfaction at each iteration.

To apply this algorithm, it is necessary to compute the gradient of the function *h*. For this, one takes advantage of the concept of *group inverse*. The reader is referred to Kirkland and Neumann (2012) for a thorough presentation of this notion.

### Definition 6.1

Assume that $B \in \mathbb {C}^{n \times n}$ is a complex singular matrix, and that its eigenvalue 0 is semisimple, that is, its algebraic and geometric multiplicities coincide. Then, the *group inverse* of *B*, denoted $B^{\#}$, is the unique matrix $X \in \mathbb {C}^{n \times n}$ such that

$$(i)~ BXB = B;\qquad (ii)~ XBX = X;\qquad (iii)~ BX = XB. $$

The following technical result shows how to compute the gradient $\nabla h(\Lambda )$ of the function h(Λ).

### Lemma 6.3

Assume *D* is irreducible. Moreover, for any $\Lambda \in \mathbb {R}^n_+$, let $Q(\Lambda ):= h(\Lambda )I - A(a+\gamma x^*_s(\Lambda ))$, and note $Q^\#(\Lambda )$ its group inverse. Then, for all $\Lambda \gg 0_n$ and i=1,…,n,

31 $$\begin{aligned} \big (\nabla h(\Lambda )\big )_i = \gamma \sum \limits _{p = 1}^n \mu _{2,p} \left( J^s \right) ^{-1}_{pi} \left( \sqrt{\frac{b_p }{\big (a_p + \gamma x^*_{s,p}(\Lambda )\big ) \mu _{2,p}}} - 1\right) ^+ \left( I- Q(\Lambda ) Q^\#(\Lambda ) \right) _{pp}. \end{aligned}$$

### Proof

See Appendix G. □

To compute the group inverse $Q^{\#}(\Lambda )$ of the matrix Q(Λ), one uses the formula provided in (Kirkland and Neumann, 2012, Remark 2.5.3). This formula is stated therein for matrices written in the form s(A)I-A, for *A* a non-negative and irreducible matrix. One shows easily that it remains valid for an irreducible Metzler matrix *A*.

In Lemma 6.3, the gradient of h(Λ) is given for $\Lambda \gg 0_n$, which ensures $x^*_s(\Lambda ) \gg 0_n$ by Theorem 4.2. Since $a + \gamma x^*_s(\Lambda ) \gg 0_n$, *h* is then differentiable with respect to Λ by Theorem 5.3. We are only interested in strictly positive values of $\Lambda _i$ for $i \in \mathcal {C}^s$, since the variables corresponding to indices in $\{1,\ldots ,n\}\setminus \mathcal {C}^s$ are of course discarded and admissible points for the interior-point algorithm must strictly satisfy the inequality constraints.

## Numerical Exploration of Different Scenarios

In this section, we consider the SIT control of the oriental fruit fly, *Bactrocera dorsalis*, by exploring different scenarios. Typical values for local parameters are provided in Table 1.

In Table 1, the competitiveness parameter γ is set to 0.39, reflecting that sterile insects are generally less competitive than wild insects in mating (Dyck et al. 2021; Dumont 2025). Notice that a higher γ reduces the number of insects that need to be released. Some treatments can enhance the competitiveness of the sterile insects. For example, when releasing sterile males of *Bactrocera dorsalis*, the male competitiveness parameter can be increased up to 1, notably through treatment with methyl eugenol before the releases (Ji et al. 2013).

Regarding the dispersal parameters $d_{ij}$, no data appear to be available in the literature. However, it is likely that mark–release–recapture experiments could provide such estimates (Jang et al. 2017; Shelly and Edu 2010). We assume here that the dispersal rates of sterile and wild insects are identical, i.e.,

$$ D^s = D. $$

**Table 1:** Parameter values for *Bactrocera dorsalis*. When mass trapping (MT) is applied, it is assumed to increase the natural mortality rates of both wild and sterile insects by 50%

**Symbol**	**Description**	**Value**	**Unit**	**Reference**
$b_i$	Adult birth rate	6.60	${\hbox {day}}^{-1}$	Ekesi et al. (2006)
$\mu _{1,i}$	Wild insect death rate	1/80.75	${\hbox {day}}^{-1}$	Ekesi et al. (2006)
$\rho _i$	Wild insect additional death rate with MT	0.5/80.75	${\hbox {day}}^{-1}$	Chosen
$\mu _{2,i}$	Wild insect density death rate	0.001	${\hbox {Ind}}^{-1}$ ${\hbox {day}}^{-1}$	Chosen
$\mu _{s,i}$	Sterile insect death rate	2/86.4	${\hbox {day}}^{-1}$	Dumont (2025)
$\rho _{s,i}$	Sterile insect additional death rate with MT	1/86.4	${\hbox {day}}^{-1}$	Chosen
γ	Sterile insect competitive parameter	0.39	–	Dumont (2025)

In this section, we display solutions of Problem (29) in different configurations, assuming that the release rates can be arbitrarily large in the release patches, that is, $\mathcal {C}^s_I = \mathcal {C}^s$, with the notations introduced in Definition 4.1. Throughout this exploration, we take the price vector to be $\pi = 1_n$, i.e., the total number of sterile insects to be released daily to eliminate the wild population is minimised. We also set $\alpha = 10^{-5}$ ${\hbox {days}}^{-1}$. The optimal release rate in each release patch is rounded up to the nearest integer.

We also assume guaranteed natural mating success in each patch ($a = 0_n$), as no empirical data are currently available. Under this worst-case scenario, by Remark 3.4, the resulting optimal release rates ensure elimination regardless of the actual value of *a*, providing robustness to the results with respect to uncertainties on the value of *a*. The impact of the Allee parameter *a* on the release duration $\tau (x_0,x_{s,0})$, defined in Definition 5.1, is studied in Sect. 7.1.3.

A three-patch example is first studied (Sect. 7.1), followed by a more complex seven-patch network (Sect. 7.2).

### Three-Patch Model

**Fig. 2:**
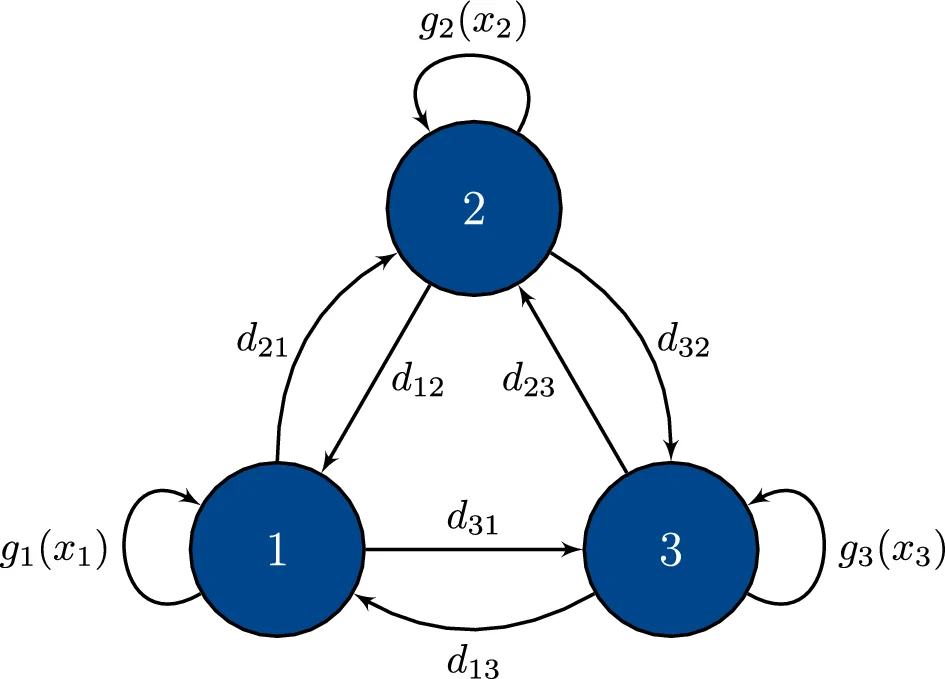
3-patch flow diagram

In this section, we assume that the network is composed of three patches (n=3), as illustrated in Fig. 2.

For parameters identical in every patch and symmetric dispersal, the natural persistence equilibrium ${x_+(0)}_i$ remains the same regardless of the dispersal coefficients (Elbetch et al. 2021). Thus, its value coincides with the persistence equilibrium obtained in the absence of dispersal, given by Theorem 3.1:

32 $$\begin{aligned} {x_+(0)}_i = 6587.62, \quad i = 1,\ldots ,n. \end{aligned}$$

#### Effects of Dispersal Rates and Density-Dependent Mortality Rates

In this section, the impact of dispersal parameters and density-dependent mortality rates on the SIT strategy is investigated, using the parameter values provided in Table 1, and identical dispersal rates between connected patches.

**7.1.1.1 Interconnected patches**

The SIT strategies associated with different release patches $\mathcal {C}^s$ are presented in Table 2 and Table 3, for dispersal coefficients all equal to 0.02 and 0.01, respectively. In these tables, the last row represents the optimal total number of sterile insects to release daily to ensure elimination of the wild population.

Since identical dispersal rates is assumed between all patches and the local parameters are identical in every patch, the outcome depends only on the number of release patches.

Even if the wild insect persistence equilibrium is the same regardless of the dispersal coefficients, by comparing Table 2 and Table 3, the critical release rate increases as the dispersal parameters decrease, except when the releases occur in all patches. In that case, both the allocation and the total number of insects released per day remain unchanged, regardless of the dispersal coefficients. As expected, the best strategy is to release the sterile insects in all three patches.

However, while our simulations show all optimal release strategies, it is important to discuss their usefulness in the field. For instance, for practical purposes, it may be better to release in one patch only: the amount of sterile insects to release is larger but it requires only one team in the field to carry out the releases, except e.g. if the three patches are sufficiently close to be visited the same day

**Table 2:** Estimates of the critical (total) release rate and the optimal release strategy for the 3-patch SIT control with $d_{ij}= d^s_{ij}= 0.02, i,j = 1,2,3, i \ne j$

$\mathcal {C}^s$	{1}	{2}	{3}	{1,2}	{1,3}	{2,3}	{1,2,3}
$\left( \Lambda _i^*\right)$	(1468,–,–)	(–,1468,–)	(–,–,1468)	(713,713,–)	(713,–,713)	(–,713,713)	(359,359,359)
$\sum _{i=1}^3 \Lambda _i^*$	1468	1468	1468	1426	1426	1426	1077

**Table 3:** Estimates of the (total) critical release rate and the optimal release strategy for the 3-patch SIT control with $d_{ij}=d^s_{ij}=0.01, i,j = 1,2,3, i \ne j$

$\mathcal {C}^s$	{1}	{2}	{3}	{1,2}	{1,3}	{2,3}	{1,2,3}
$\left( \Lambda _i^*\right)$	(1881,–,–)	(–,1881,–)	(–,–,1881)	(921,921,–)	(921,–,921)	(–,921,921)	(359,359,359)
$\sum _{i=1}^3 \Lambda _i^*$	1881	1881	1881	1842	1842	1842	1077

We now consider the case where one patch (say, Patch 2) has a lower density-dependent mortality rate ($\mu _{2,2} = 0.0005$), corresponding to a higher carrying capacity. The natural persistence equilibria are reported in Table 4, and the SIT strategies in Table 5 and Table 6, for dispersal coefficients equal to 0.02 and 0.01, respectively. As expected, the natural equilibrium as well as the number of sterile insects to be released daily are larger than in the case of patches with equal density-dependent mortality rates (compare with Table 2 and Table 3), since the total carrying capacity is larger. However, unlike the equal density-dependent mortality rate scenario—where the persistence equilibrium is independent of dispersal—the equilibrium here varies with dispersal rates.

A patch with a lower density-dependent mortality rate also changes the allocation strategy. For any number of release patches, the best strategy is always to release more sterile insects in the patch with lower density-dependent mortality rate (Patch 2). This is due to the fact that Patch 2 has the largest pest population at equilibrium.

If releases occur only in Patch 2, the corresponding release rate remains the same as in the equal density-dependent mortality rate case, resulting in the same amount of sterile insects to be released daily (compare the second columns of Tables 5 and 6 with Tables 2 and 3). Moreover, according to Table 5 and Table 6, releasing in two patches, say $\{1,2\}$ or $\{2,3\}$, yields nearly the same outcome as releasing in Patch 2 alone. Therefore when a patch has a lower density-dependent mortality rate and the two others are identical, the best strategy—if the releases cannot occur in all patches—is to release only in the patch with lower density-dependent mortality rate, which avoids deploying two teams without affecting significantly the release rate.

As in the equal density-dependent mortality rate scenario, the required release rate increases as dispersal decreases, except when all patches are treated. In that case, the total release rate remains nearly unchanged, as in the case of patches with equal density-dependent mortality rate; however, the allocation now varies with dispersal.

**Table 4:** 3-patch model with $\mu _{2,2} = 0.0005$: persistence equilibrium of the wild population versus dispersal rates

Dispersal rate between all patches	Natural persistence equilibrium
0.02	(6607.38, 13135.47, 6607.38)
0.01	(6597.56, 13155.29, 6597.56)

**Table 5:** Estimates of the critical (total) release rate and the optimal release strategy for the 3-patch SIT control with $d_{ij}= d^s_{ij}=0.02, i,j = 1,2,3, i \ne j$ and $\mu _{2,2} = 0.0005$

$\mathcal {C}^s$	{1}	{2}	{3}	{1,2}	{1,3}	{2,3}	{1,2,3}
$\left( \Lambda ^*_i\right)$	(2850,–,–)	(–,1469,–)	(–,–,2850)	(93,1356,–)	(1425,–,1425)	(–,1356,93)	(70,1291,70)
$\sum _{i=1}^3 \Lambda _i^*$	2850	1469	2850	1449	2850	1449	1431

**Table 6:** Estimates of the critical (total) release rate and the optimal release strategy for the 3-patch SIT control with $d_{ij}=d^s_{ij}=0.01, i,j = 1,2,3, i \ne j$ and $\mu _{2,2} = 0.0005$

$\mathcal {C}^s$	{1}	{2}	{3}	{1,2}	{1,3}	{2,3}	{1,2,3}
$\left( \Lambda ^*_i\right)$	(3683,–,–)	(–,1881,–)	(–,–,3683)	(838,1004,–)	(1842,–,1842)	(–,1004,838)	(213,1007,213)
$\sum _{i=1}^3 \Lambda _i^*$	3683	1881	3683	1842	3684	1842	1433

##### Remark 7.1

The carrying capacity affects the release strategy here, whereas in our previous work (Bliman et al. 2025), where a logistic-type local growth function was considered, it did not influence the optimal additional mortality rate induced by mass trapping (MT). This is because the release of sterile insects induces a strong Allee effect in the model.

**7.1.1.2 Chain Structure**

**Fig. 3:**
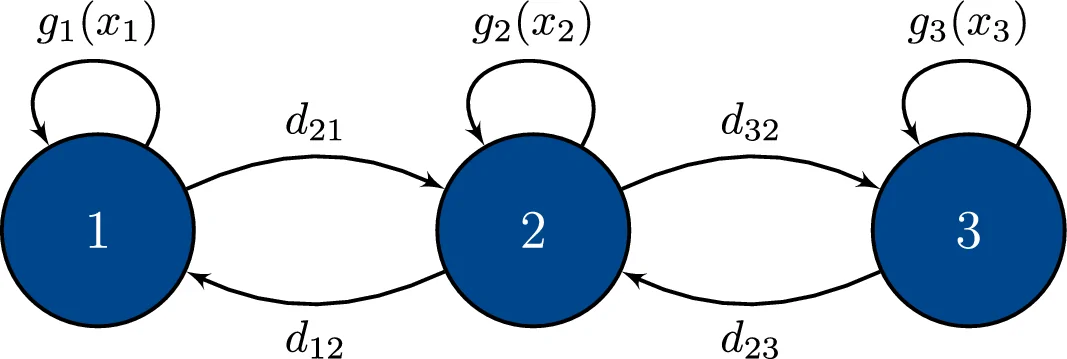
3-patch flow diagram with a chain structure

In this section, we assume that $d_{13} = d_{31} = 0$, so that the network has a chain structure, as illustrated in Fig. 3.

When the patches have equal density-dependent mortality rates ($\mu _{2,i}=0.001$, i=1,2,3) and the dispersal is symmetric, the persistence equilibrium of the wild insect population coincides with that in the absence of dispersal (see (32)). The SIT strategies are listed in Table 7 and Table 8, for the dispersal coefficients equal to 0.02 and 0.01, respectively. If releases are allowed in all three patches, in Patches $\{1,3\}$, or in Patch 2—cases that reflect the model symmetry—the SIT strategy remains unchanged compared to the fully connected scenario (see Table 2 and Table 3). In the remaining configurations, the strategy is altered and larger releases are required.

Notice that releasing in two patches, or in Patch 2 only, does not substantially affect the number of sterile insects to be released daily. However, in the two-patch release case, only the releases in Patches $\{1,3\}$ are realistic. In particular, releasing in Patch 2 alone is nearly equivalent to releasing in Patches $\{1,2\}$ or $\{2,3\}$, since Patch 2 is directly connected to the two other patches. Given the limited gain achieved by releasing in two patches instead of one, it may be sufficient to adopt the slightly suboptimal strategy of releasing in Patch 2 only, thereby avoiding the need to deploy two teams in the field.

**Table 7:** Estimates of the critical (total) release rate and the optimal release strategy for the 3-patch SIT control when the network has a chain structure, with $d_{12} = d_{21} = d_{23} = d_{32} =0.02$

$\mathcal {C}^s$	{1}	{2}	{3}	{1,2}	{1,3}	{2,3}	{1,2,3}
$\left( \Lambda ^*_i\right)$	(3117,–,–)	(–,1468,–)	(–,–,3117)	(16,1448,–)	(713,–,713)	(–,1448,16)	(359,359,359)
$\sum _{i=1}^3 \Lambda _i^*$	3117	1468	3117	1464	1426	1464	1077

**Table 8:** Estimates of the critical (total) release rate and the optimal release strategy for the 3-patch SIT control when the network has a chain structure, with $d_{12} = d_{21} = d_{23} = d_{32} =0.01$

$\mathcal {C}^s$	{1}	{2}	{3}	{1,2}	{1,3}	{2,3}	{1,2,3}
$\left( \Lambda ^*_i\right)$	(6185,–,–)	(–,1881,–)	(–,–,6185)	(8,1870,–)	(921,–,921)	(–,1870,8)	(359,359,359)
$\sum _{i=1}^3 \Lambda _i^*$	6185	1881	6185	1878	1842	1878	1077

When Patch 2 has a lower density-dependent mortality rate $(\mu _{2,2} = 0.0005)$, the natural persistence equilibrium of the wild population is still the same as in the fully connected case (see Table 4). The SIT strategies for dispersal coefficients of 0.02 and 0.01 between connected patches are reported in Table 9 and Table 10, respectively. A comparison of Table 9 (Table 10) with Table 7 (Table 8) shows that, unlike in the fully connected case, reducing the density-dependent mortality rate in Patch 2 only alters the SIT strategy when releases occur in both Patches 1 and 3.

Notice that when releases can occur in at most two patches, as in the case with identical patches, the best strategy is to release only in the patch with lower density-dependent mortality rate (Patch 2), especially when dispersal increases.

**Table 9:** Estimates of the critical (total) release rate and the optimal release strategy for the 3-patch SIT control when the network has a chain structure, with $d_{12} = d_{21} = d_{23} = d_{32} =0.02$ and $\mu _{2,2} = 0.0005$

$\mathcal {C}^s$	{1}	{2}	{3}	{1,2}	{1,3}	{2,3}	{1,2,3}
$\left( \Lambda ^*_i\right)$	(3123,–,–)	(–,1469,–)	(–,–,3123)	(14,1452,–)	(1425,–,1425)	(–,1452,14)	(70,1291,70)
$\sum _{i=1}^3 \Lambda _i^*$	3123	1469	3123	1466	2850	1466	1431

**Table 10:** Estimates of the critical (total) release rate and the optimal release strategy for the 3-patch SIT control when the network has a chain structure, with $d_{12} = d_{21} = d_{23} = d_{32} =0.01$ and $\mu _{2,2} = 0.0005$

$\mathcal {C}^s$	{1}	{2}	{3}	{1,2}	{1,3}	{2,3}	{1,2,3}
$\left( \Lambda ^*_i\right)$	(6185,–,–)	(–,1881,–)	(–,–,6185)	(8,1870,–)	(1842,–,1842)	(–,1870,8)	(213,1007,213)
$\sum _{i=1}^3 \Lambda _i^*$	6185	1881	6185	1878	3684	1878	1433

To conclude this section, when releasing in all patches is not feasible, the best strategy is to focus mainly on the patch with lower density-dependent mortality rate. In particular, when this patch is well connected to the others, then releasing only in this single patch may be the best strategy. Indeed, releasing in two patches does not substantially decrease the total critical release rate, but requires the deployment of two teams on the ground, increasing the cost of the SIT programme.

#### Combining SIT with Mass Trapping (MT)

In this section, the effect of mass trapping (MT) on the SIT strategy is investigated for a fully connected network, as in Fig. 2. Recall that traps also capture sterile insects, and the corresponding additional mortalities are provided in Table 1. Each dispersal coefficient is set to 0.02.

In practice, producers use MT against *Bactrocera dorsalis*. In the following numerical simulations, traps are assumed to be removed in the patches where sterile insects are released, because MT can negatively impact the sterile insects. However, trap removal can be costly, especially when large areas are involved.

The release strategies in Table 2, obtained without MT, will serve as reference scenario. The corresponding strategies with MT in the patches where sterile insects are not released are shown in Table 11. Comparing this table with Table 2 shows that trap deployment requires higher release rates to achieve elimination. When releasing in a single patch, applying MT increases the critical release rate by 35.5% (1989 vs. 1468). When releasing in two patches, applying MT increases it by 24.0% (1768 vs 1426). Such increases may pose challenges when sterile insect production is limited.

**Table 11:** Estimates of the critical (total) release rate and the optimal release strategy for the 3-patch SIT control depending on the release patches $\mathcal {C}^s$ when MT is applied in the remaining patches

$\mathcal {C}^s$	{1}	{2}	{3}	{1,2}	{1,3}	{2,3}
$\left( \Lambda ^*_i\right)$	(1989,–,–)	(–,1989,–)	(–,–,1989)	(884,884,–)	(884,–,884)	(–,884,884)
$\sum _{i=1}^3 \Lambda _i^*$	1989	1989	1989	1768	1768	1768

To conclude this section, regarding release rates, the additional sterile mortality induced by MT outweighs the gain from increased wild mortality. However, we show in the next section that, when a strong Allee effect is naturally present in every patch and for fixed release rates, MT may reduce the release duration $\tau \big (x_+(a),0\big )$ and thus the total number $\Sigma _s\big (x_+(a),0\big )$ of sterile insects released until entering the uncontrolled basin of attraction of the origin.

#### Strong Allee Effect and Sterile Insect Release Duration

In the previous simulations, mating success in each patch was assumed to be guaranteed, i.e., $a = 0_n$, which implies that sterile insects must be released indefinitely (Anguelov et al. 2020); otherwise, the wild population would return to its persistence equilibrium. When mating conditions are imperfect in each patch, the SIT can be stopped after a finite time needed to enter the basin of attraction of the origin of the uncontrolled model (6), such that the population will decay till elimination (Theorem 5.5).

In the following simulations, we use as a baseline the optimal critical release rate obtained in the case $a = 0_n$, denoted by $\Lambda _0^*$. Recall that this release rate guarantees elimination for any value of $a \in \mathbb {R}^n_+$. Compared to the previous sections, the index 0 in $\Lambda _0^*$ emphasises that the critical release rates considered here correspond to the case where no strong Allee effect is present in any patch.

In field applications, mass releases are performed. By Theorem 5.3 and corollary 3.10, any release rate Λ such that $\Lambda \ge \Lambda ^*_0$ drives the wild population to elimination, regardless of $a\in \mathbb {R}^n_+$. We express here the release values as multiples of $\Lambda _0^*$, namely $\Lambda = p \times \Lambda _0^*$ for p=2,5,10 and 30. For each value of Λ, we examine how the Allee parameter *a* impacts the release duration $\tau \big (x_+(a),0\big )$ and the total number $\Sigma _s\big (x_+(a),0\big )$ of sterile insects released until entering the uncontrolled basin of attraction of the origin (see Definition 5.1). We also analyse how these two indicators vary with different release rates Λ.

As said in the beginning of Sect. 7.1.2, numerical simulations are performed with dispersal coefficients set to 0.02.

**Fig. 4:**
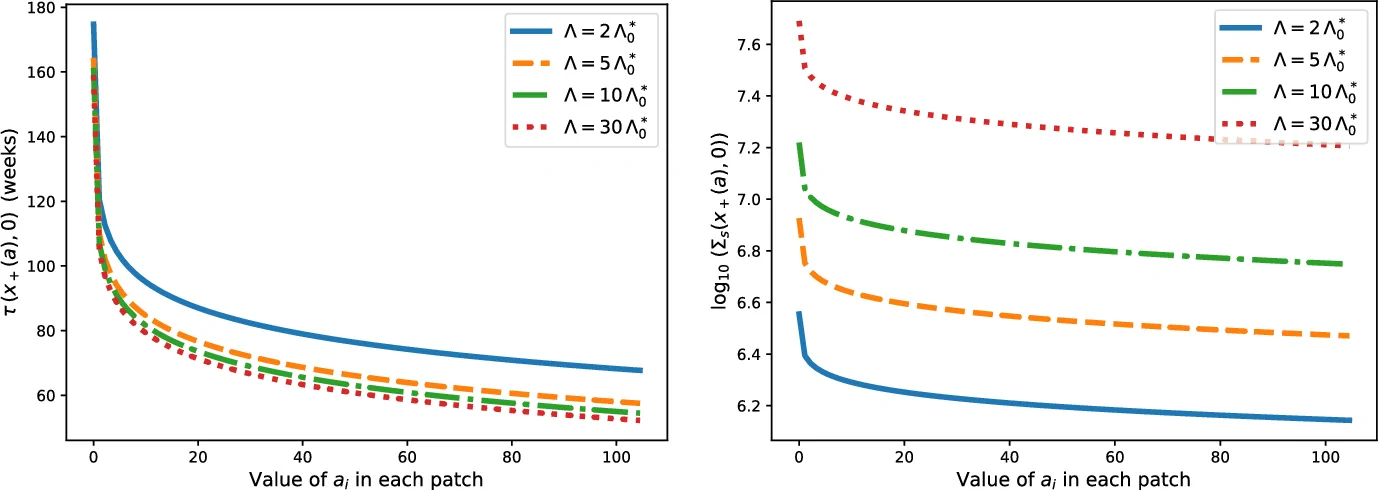
3-patch SIT control with $\mathcal {C}^s=\{2\}$: **(a)** $\tau (x_+(a),0)$ and **(b)** $\Sigma _s(x_+(a),0)$ as functions of the Allee parameter. Both $\tau \big (x_+(a),0\big )$ and $\Sigma _s\big (x_+(a),0\big )$ decrease with respect to both the Allee parameter and the release rates

**Fig. 5:**
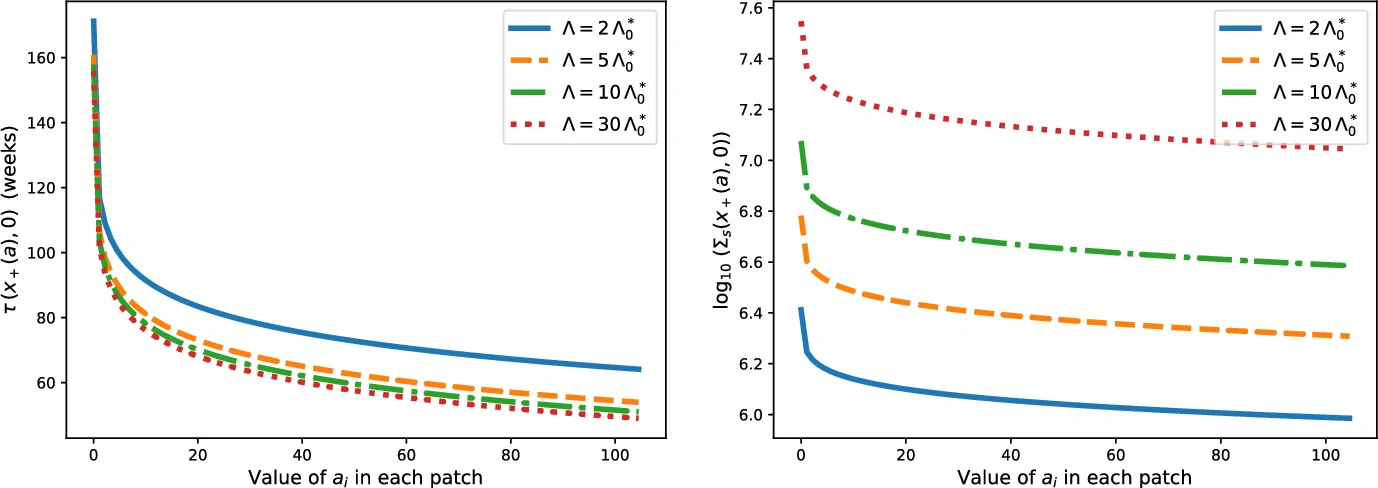
3-patch SIT control with $\mathcal {C}^s=\{1,2,3\}$: **(a)** $\tau (x_+(a),0)$ and **(b)** $\Sigma _s(x_+(a),0)$ as functions of the Allee parameter. By comparing with Fig. 4, releasing in all three patches reduces $\Sigma _s\big (x_+(a),0\big )$ relative to the one-patch release case

Figures 4(a) and 5(a), show that $\tau \big (x_+(a),0\big )$ decreases rapidly as $a_i$ increases in all patches, and also decreases with respect to p=2, 5 10 and 30. However, the release duration curves for p=5, p=10 and p=30 are very close, and thus $\Sigma _s\big (x_+(a),0\big )$ grows dramatically as *p* increases (see Fig. 4(b) and Fig. 5(b)). These observations suggest the existence of a threshold release rate, beyond which further increases provide no practical advantage. The best result for $\Sigma _s\big (x_+(a),0\big )$ is obtained for $\Lambda = 2 \Lambda _0^*$.

As shown earlier in Tables 7–10, the critical release rate decreases as the number of release patches increases. Moreover, comparing Fig. 4(a) and Fig. 5(a) shows that the release durations in the one-patch and three-patch release cases are similar. This implies that, due to the lower critical release rates in the three-patch release case, $\Sigma _s\big (x_+(a),0\big )$ is significantly reduced when releases are carried out in three patches (compare Fig. 4(b) and Fig. 5(b)).

**Fig. 6:**
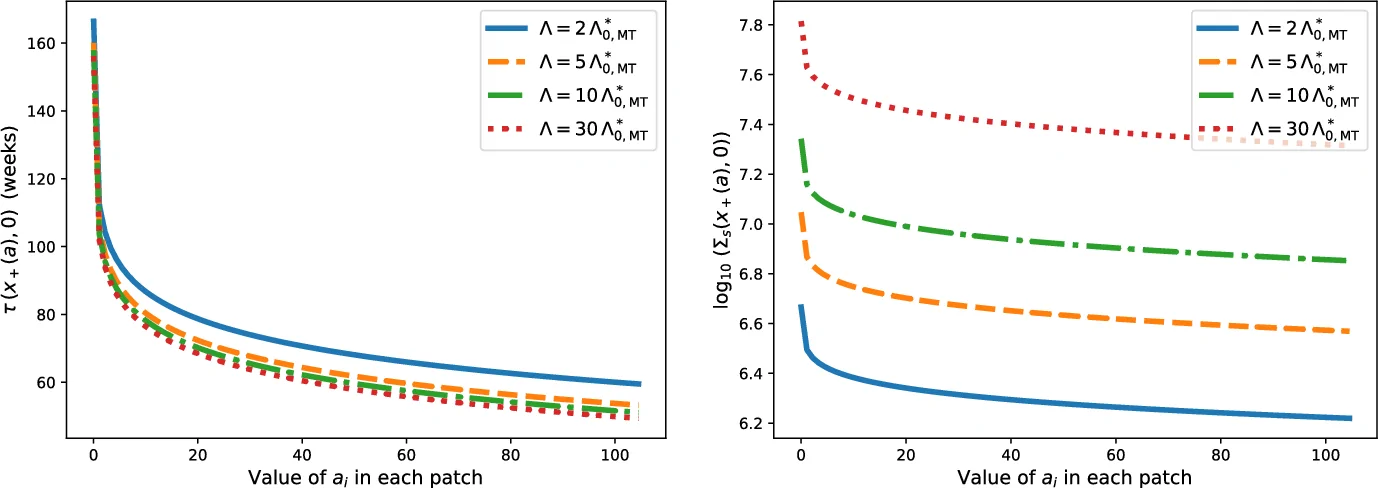
3-patch SIT control with $\mathcal {C}^s=\{2\}$: **(a)** $\tau (x_+(a),0)$ and **(b)** $\Sigma _s(x_+(a),0)$ as functions of the Allee parameter. In contrast to Fig. 4, the release rates Λ are chosen as multiples of the critical release rate obtained with MT in Patches $\{1,3\}$

**Fig. 7:**
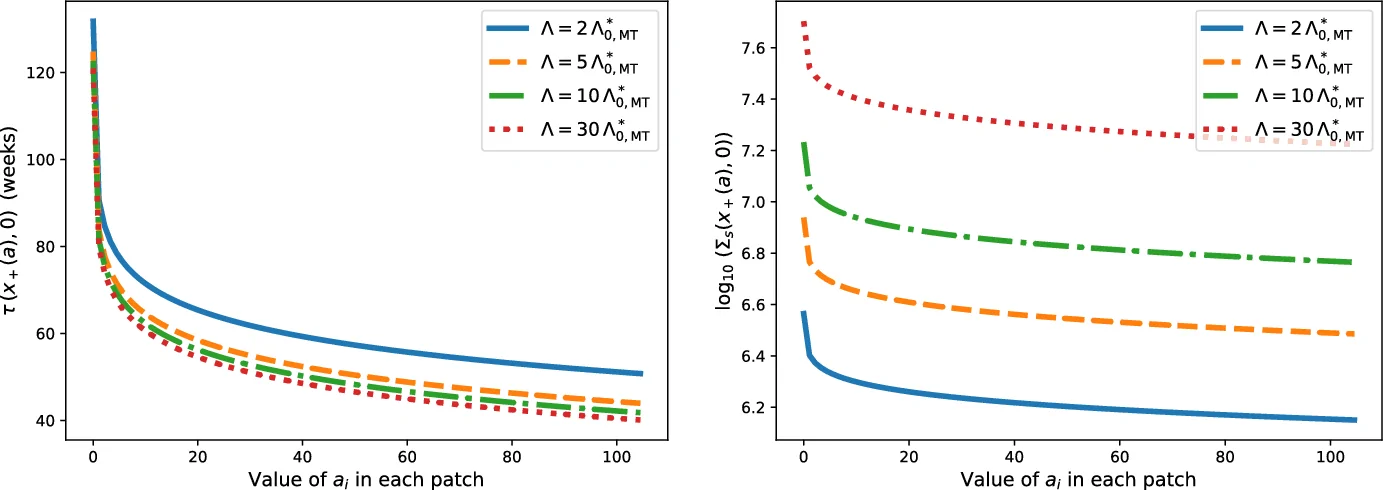
3-patch SIT control with $\mathcal {C}^s=\{2\}$ and MT in Patches {1,3}: **(a)** $\tau (x_+(a),0)$ and **(b)** $\Sigma _s(x_+(a),0)$ as functions of the Allee parameter. By comparing with Fig. 6, applying MT reduces both $\tau \big (x_+(a),0\big )$ and $\Sigma _s\big (x_+(a),0\big )$ relative to the SIT-only case

We now investigate the impact of mass trapping (MT) on the release duration. As shown in Sect. 7.1.2, the use of MT induces a higher critical release rate required for elimination compared to the SIT-only case. To enable a meaningful comparison of release durations between the SIT-only case and the SIT-MT case, we consider identical release rates, using as a reference the critical release rates obtained with MT, which are larger and denoted by $\Lambda ^*_{0,\textrm{MT}}$ (see Table 11). By comparing Fig. 7(a) and Fig. 6(a), applying MT reduces $\tau \big (x_+(a),0\big )$, and consequently $\Sigma _s\big (x_+(a),0\big )$ (compare Fig. 7(b) and Fig. 6(b)).

In all simulations, MT is stopped at the same time as the releases, i.e., the entrance time into the basin of attraction of the extinction equilibrium of the uncontrolled model (6) is evaluated by setting $\rho _i = \rho _{s,i} = 0$ in (6), although trap removal can be costly. In practice, traps may be maintained beyond the release period. Here, they are removed simultaneously with the end of the releases to consider a conservative scenario; maintaining them would in fact reduce the required release duration.

### Seven-Patch Model and Impact of the Network Configuration

We consider in this section examples of larger networks, with seven patches (n=7). The same configurations were examined in Bliman et al. (2025) with mass trapping (MT) control only.

In the field, with numerous areas, it may be impossible or too costly to deploy teams to release sterile insects in all patches. Therefore, for each network configuration, three scenarios are examined, with the number of sterile-release patches set consecutively to 1, 2, and 3. This amounts to solving the minimisation problem (29) with an additional constraint on the number of positive entries of the release vector Λ, i.e., for k=1,2,3, solving the following problem:

33 $$\begin{aligned} \begin{array}{ll} \text {minimise} & \textbf{1}_n^\top \Lambda \\ \text {subject to} & \Lambda ~\overline{\Lambda }\text {-admissible} \\ & h(\Lambda ) \le -10^{-5} \\ & \#\{ i \;:\; \Lambda _i > 0 \} \le k, \end{array} \end{aligned}$$

where the function *h* is defined in (30), and $\#\{i: \Lambda _i > 0 \}$ denotes the number of positive components of Λ. The combinations of *k* patches that minimise the total release rate are then identified as the optimal combinations of release patches.

The objective of this section is to assess the impact of the network’s spatial structure on the optimal selection of sterile-release patches. To this aim, we consider two different networks. For each of them, we first consider the unconstrained scenario, in which releases are allowed in all patches $(\mathcal {C}^s = \mathcal {C}^s_I = \{1,\ldots ,7\})$. We then examine how prohibiting releases in certain patches, as may occur when landowners do not allow them, can significantly increase the number of sterile insects required. Finally, we study the application of MT in specific areas and its impact on the optimal critical release rate and the release duration.

To see the net effect of the network structure, we choose uniform local parameters across all patches, with parameters in each patch as in Table 1. The dispersal parameters for the wild and sterile insects are assumed identical ($D^s = D$), and the dispersal coefficients between connected patches are set to 0.02.

#### Configuration 1

**Fig. 8:**
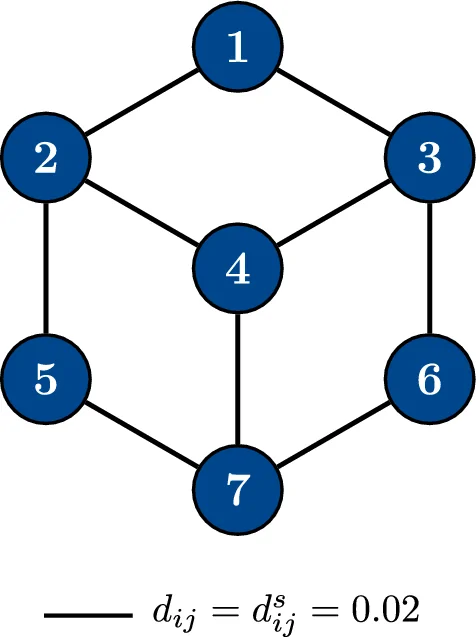
A network configuration with seven patches

Let us begin with the example illustrated in Fig. 8, where lines connecting two patches illustrate bidirectional and symmetric dispersal.

The optimal combinations of *k* release patches are presented in Table 12, in the absence of MT, for k=1,2 and 3. The corresponding optimal release rates are also reported in the table: first without MT (SIT-only case), and then with MT in the remaining patches (SIT-MT case).

Without MT, releasing insects in a single patch requires a total daily release rate of 4259 individuals, which is 29.4% more than when two patches are available for release (3291 individuals). Releasing in two patches results in only a 7.0% higher total daily release compared with three patches (3075 individuals). Applying SIT in 3 patches rather than 2 thus results in limited savings in terms of sterile insect production, to be compared with the greater organisational complexity.

**Fig. 9:**
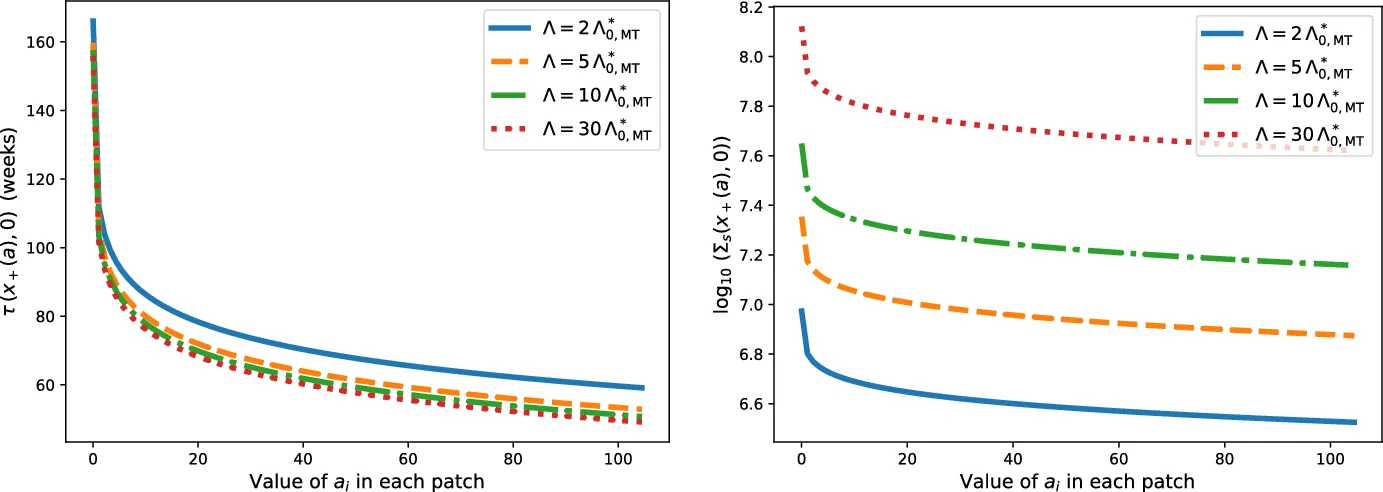
7-patch SIT control for Configuration 1 with $\mathcal {C}^s=\{2,3,7\}$: **(a)** $\tau (x_+(a),0)$ and **(b)** $\Sigma _s(x_+(a),0)$ as functions of the Allee parameter

**Fig. 10:**
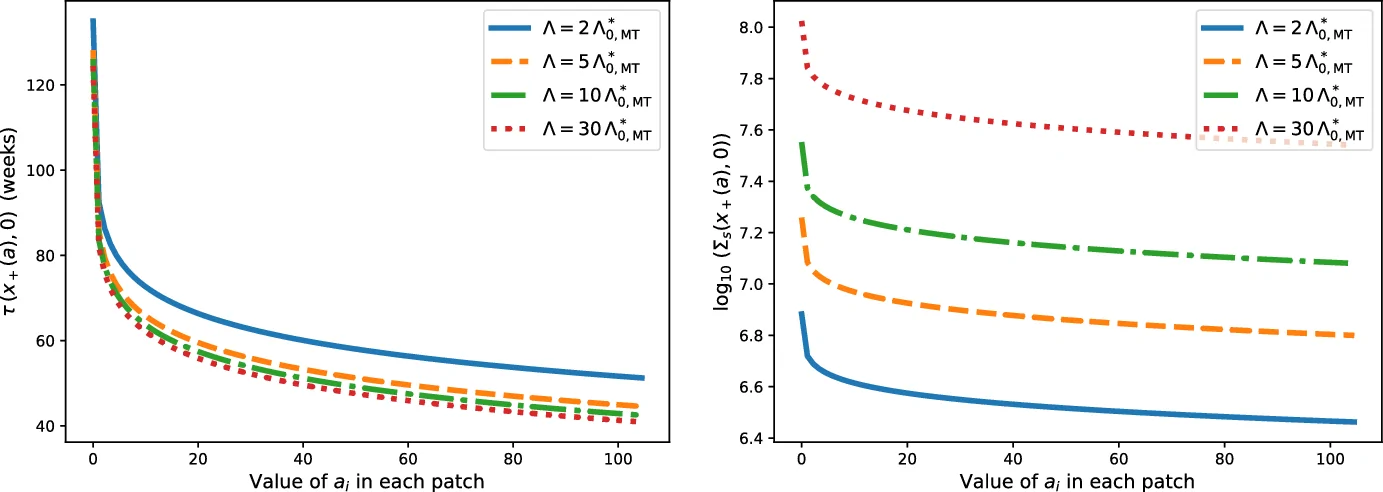
7-patch SIT control for Configuration 1 with $\mathcal {C}^s=\{2,3,7\}$ and MT in the remaining patches: **(a)** $\tau (x_+(a),0)$ and **(b)** $\Sigma _s(x_+(a),0)$ as functions of the Allee parameter. By comparing with Fig. 9, applying MT reduces both $\tau \big (x_+(a),0\big )$ and $\Sigma _s\big (x_+(a),0\big )$ relative to the SIT-only case

When MT is applied outside the release patches, the daily release rates required for elimination increase according to Table 12. However, for fixed release rates, $\tau \big (x_+(a),0\big )$ decreases (compare Fig. 10(a) with Fig. 9(a), page, for $\mathcal {C}^s=\{2,3,7\}$), leading to a reduction in $\Sigma _s\big (x_+(a),0\big )$ (compare Fig. 10(b) with Fig. 9(b)). Further numerical simulations, not reported here, show that increasing the number of patches where MT is applied leads to higher optimal critical release rates but, for fixed release rates, to shorter release durations and thus lower total quantities $\Sigma _s\big (x_+(a),0\big )$.

**Table 12:** Optimal combinations of release patches (in green) depending on the number *k* of release sites. The amounts of sterile insects released daily are shown with and without MT in the remaining patches (in blue)

*k*	Optimal release patch combinations	$\sum _{i=1}^7 \Lambda _i^*$	
		SIT-only	SIT-MT
1		4259	6687
2		3291	4503
3		3075	4044

Restricting the number of patches on which SIT is applied is not the only logistical preoccupation: for various reasons, some patches may be unavailable. This constraint can alter the optimal intervention strategy. That is why numerical simulations can be useful to explore all possible intervention strategies in order to help the field experts and increase the SIT efficacy.

The (optimal) release strategies when Patches {4,5,6} are unavailable for releases ($\mathcal {C}^s= \mathcal {C}^s_I = \{1,2,3,7\}$) are presented in Table 13. Three categories of patches are considered, with the corresponding colours shown in the table. Patches where releases cannot be achieved are shown in red, while the set of optimal patches – among those where releases are permitted – is shown in green. In the remaining patches, shown in blue, MT may or may not be applied.

When the releases are limited to a single patch, the best strategy is to release insects in Patch 1. This placement necessitates a daily release of 6757 sterile insects, representing a 58.7% increase compared to the unconstrained scenario, in which the optimal patch is the central Patch 4 (see Table 12).

If intervention is restricted to at most two patches (k=2), among the three optimal strategies identified in Table 12 for the unconstrained case, only one remains feasible: Patches $\{1,7\}$. When releases are limited to a maximum of three patches (k=3), and since the optimal combination for the unconstrained case does not involve the forbidden Patches {4,5,6} (see Table 12), the best strategy remains unchanged.

Naturally, in the absence of MT, the daily release rates for k=2 or k=3 remain identical to those of the unconstrained scenario since the optimal release patches are unchanged. Differences arise only when MT is applied in patches that are neither release patches nor forbidden. In that case, the required daily release decreases relative to the unconstrained setting, since traps are deployed in fewer patches.

**Table 13:** Optimal combinations of release patches (in green) depending on the number *k* of release sites, when some patches (in red) are excluded. The amounts of sterile insects released daily are shown with and without MT in the remaining patches (in blue)

*k*	Optimal release patch combinations	$\sum _{i=1}^7 \Lambda _i^*$	
		SIT-only	SIT-MT
1		6757	9705
2		3291	3806
3		3075	3351

Also notice that when k=3 in Table 13, the release rates without MT are similar in Patches 2, 3, and 7, whereas this is no longer the case when MT (in Patch 1) is considered. In that case, the release rate in Patch 7 is significantly lower than those in Patches 2 and 3, since Patch 7 is more distant from Patch 1. However, it is important to keep in mind that these values correspond to minimal release rates ensuring elimination in all patches. If sterile insect production is sufficiently large, field experts may opt for higher release rates in Patch 7.

#### Configuration 2 – Chain Structure

Let us now consider the scenario where the graph exhibits a chain structure. This type of structure occurs when the orchards, or plots, are next to each other. In such cases, it is likely that insects will migrate from one orchard to another sequentially, without skipping any.

The optimal combinations are shown in Table 14. In particular, when sterile insects can be released in only one patch, the optimal one is the same as in Configuration 1 (Patch 4), and requires a daily release of 11847 individuals. This is 2.78 times more sterile insects to release than in Configuration 1: see Table 12. This shows the importance of the network structure in the optimal size of the releases.

When MT is applied in the remaining patches, 24026 sterile insects per day are required, which is 3.59 times more than in Configuration 1. Thus, the simultaneous use of MT and SIT may render the elimination of the wild population impossible in practice if the daily production of sterile insects is insufficient.

**Table 14:** Optimal combinations of release patches (in green) depending on the number *k* of release sites. The amounts of sterile insects released daily are shown with and without MT in the remaining patches (in blue)

*k*	Optimal release patch combinations	$\sum _{i=1}^7 \Lambda _i^*$	
		SIT-only	SIT-MT
1		11847	24026
2		5326	8426
3		3288	4328

The release strategies when sterile insects cannot be released in Patches $\{3,4,5\}$ are presented in Table 15. Notably, when sterile insects are released in only one patch, then Patch 2 or Patch 6 is optimal, but the daily release rate is extremely high compared with the unconstrained scenario (88835 vs 11847 individuals), which may be impossible in practice. When MT is applied in the remaining patches, the release rate rises to the value of 148577 individuals per day, which is substantially larger. This example highlights the important additional production cost that may be induced by the unavailability of some patches.

From table 15, we also see that without MT, releasing sterile insects in two or three patches provides the same result, so that it is sufficient to release sterile insects in Patches {2,6}.

**Table 15:** Optimal combinations of release patches (in green) depending on the number *k* of release sites, when some patches (in red) are excluded. The amounts of sterile insects released daily are shown with and without MT in the remaining patches (in blue)

*k*	Optimal release patch combinations	$\sum _{i=1}^7 \Lambda _i^*$	
		SIT-only	SIT-MT
1		88835	148577
2		5346	5574
3		5346	5432

##### Remark 7.2

In Bliman et al. (2025), we performed analogous numerical simulations for Configuration 1 and Configuration 2, but we focused on minimising the additional mortality induced by MT and for three controllable patches. Notably, for these two configurations and when the connectivity matrix is the same for wild and sterile individuals, the optimal combination of three patches for sterile insect release is exactly the same as that for the introduction of additional mortality. Further numerical simulations suggest that this is still true when $D^s$ is proportional to *D*, i.e., $D^s = \omega D$ for some ω>0. However, when the networks defined by *D* and $D^s$ are not correlated, the outcome changes significantly, and the optimal combinations for sterile insect release and for the application of MT have no reason to coincide.

### Numerical Computations of the Release Duration

In all the simulations related to release durations that were presented, the exact release durations were determined. In this section, the methodology used for these computations is presented. An alternative method, based on Theorem 5.6, is also provided. Then, we quickly compare the computation times of both methods.

To estimate the (exact) release duration, we first solve the time-varying system of ODEs (23) using the function odeint from the scipy.integrate module of the SciPy package in Python (Virtanen et al. 2020). This function relies on the LSODA solver from the ODEPACK library (Hindmarsh 1983), which automatically switches between methods for stiff and non-stiff systems (Petzold 1983). A bisection procedure is then applied along the simulated trajectory. We consider an interval $$[t_{\min }, t_{\max }]$$ and iteratively refine it. At each step, we set $$t' = (t_{\min } + t_{\max })/2$$ and evaluate the state of the system (23) at time $t^{\prime }$. This state is then used as an initial condition for the uncontrolled system (6), and we test whether the corresponding trajectory converges to the origin. If convergence occurs, we update $$t_{\max } \leftarrow t'$$, otherwise, we update $$t_{\min } \leftarrow t'$$.

With this simple estimation method, assessing the exact value of the release duration becomes computationally demanding as the number of patches increases. The formula given in Theorem 5.6 to approximate the release duration therefore offers substantial computational savings, at the price of reduced accuracy (but guaranteed overestimation). To apply this theorem, and as with estimating the exact release duration, we solve numerically the time-varying system of ODEs (23), and then apply a bisection procedure along the simulated trajectory. While, at each step of the bisection procedure, the previous approach directly checks convergence of the uncontrolled trajectory, this second method replaces this test with the condition $\Psi \left( c^{\textsf {T}} x^k \right) < -s(J_a)$, where $x^k$ denotes the state at the current iteration, and $c \gg 0_n$, and Ψ are defined in Theorem 5.6.

**Fig. 11:**
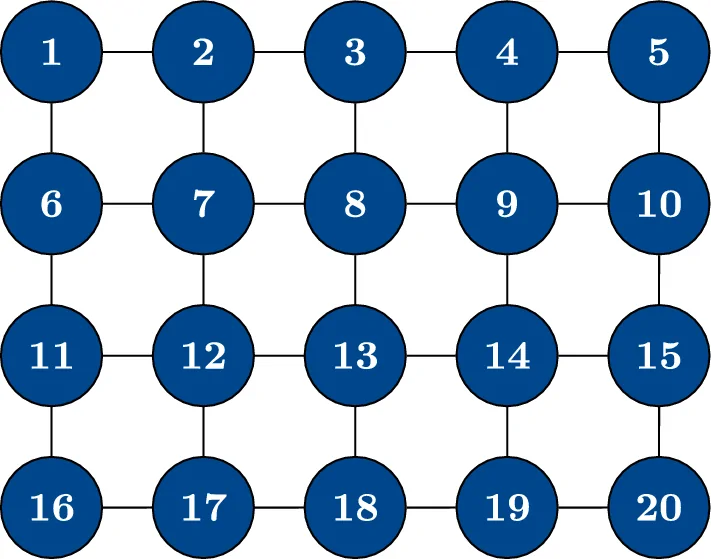
20-patch network

**Fig. 12:**
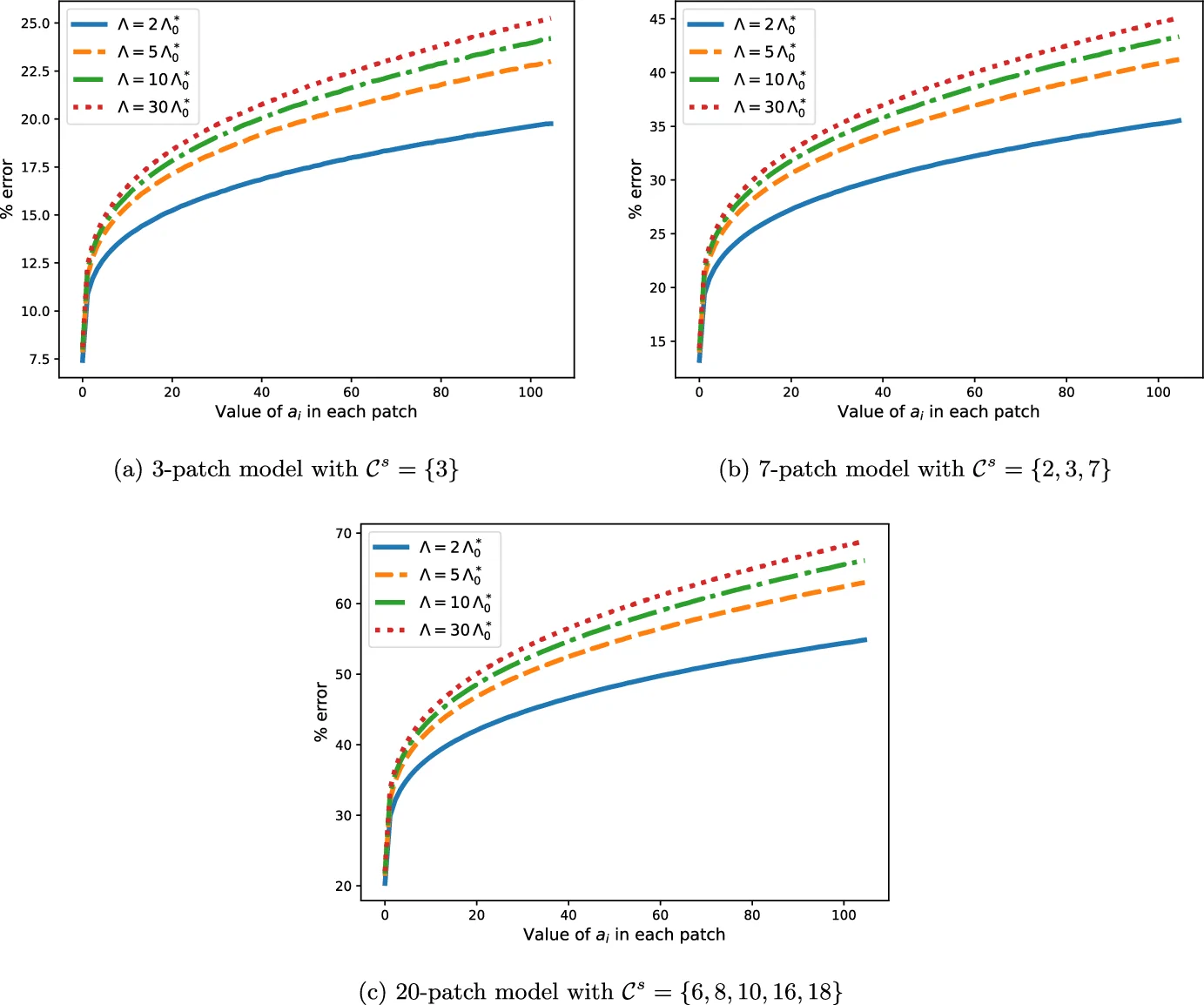
Overestimates (in percent) of $\tau \big (x_+(a),0\big )$ with Theorem 5.6, as a function of the Allee parameter. The overestimation increases with the number of patches in the network, the release rates and the Allee parameter

A summary of the computation times for the exact and estimated release durations is provided in Table 16, for the 3-patch and 7-patch (see Fig. 8) cases, as well as for a network of 20 patches with a grid structure as in Fig. 11. It is straightforward to check that the computation of the approximate release duration is, on average, 7 to 8 times faster than the exact estimate. However, as shown in Fig. 12, this gain in computational time is offset by a loss of precision in the release duration, which is overestimated in a proportion of up to 70% and increases with both the Allee parameters $a_i$ and the release rates. Moreover, a comparison of Figs. 12(a)–(c) also shows that this overestimation increases as the number of patches in the network increases.

**Table 16:** Numerical computation time for the release duration, for 96 values of $a_i \in (0,100]$ and all tested values of $\Lambda = p \times \Lambda _0^*$ with p=2,5,10,30. Computations have been made on a laptop with an Apple M3 chip and 16 Gb of RAM

Number of patches	Computational time (seconds) to estimate $\tau \big (x_+(a),0\big )$	
	Exact estimation	Estimation with Theorem 5.6
3	17.48	2.46
7	34.64	4.49
20	111.35	14.72

## Conclusion

In order to design spatial SIT strategies, an explicit metapopulation model with an arbitrary number of patches has been proposed and studied, allowing for distinct dispersal networks for wild and sterile populations. Considering constant sterile insect release rates, a sufficient condition has been established to ensure elimination of the pest/vector population under various constraints, including the presence of patches where releases are not feasible. The derived elimination condition provides a unifying criterion that combines local patch-level conditions and dispersal dynamics into a single framework. Building on this result, a convex constrained optimisation problem has been formulated and numerically solved to minimise a weighted sum of the release rates required for elimination. This framework enables the identification of efficient spatial release strategies to achieve population elimination. Among the admissible strategies, field experts may choose those that are most suitable for implementation in realistic field settings.

Through numerical simulations, we studied different scenarios, including different network structures. The results obtained highlight the importance of taking dispersal rates and network configuration into account when designing SIT strategies. In addition, from an integrated control perspective, we assessed the impact of combining mass trapping (MT) and SIT on the costs of the latter. MT also increases the sterile insect mortality, and the numerical simulations show a subsequent increase in the critical release rates required for elimination. However, for fixed release rates, MT becomes beneficial in the presence of a natural strong Allee effect in every patch, as it reduces the release duration and thus the total number of sterile insects released over the entire SIT programme. Notice that the negative effect of MT on the critical release rates may be limited if the sterile insects are treated before the releases in order to reduce their susceptibility to the traps. For instance, sterile males reared on a methyl eugenol (ME)-based diet exhibit reduced attraction to ME-based traps (Wee and Rosli 2025).

Overall, our results show that effective strategies must consider not only critical release rates required to eliminate the population, but also release duration, total releases until elimination, and logistical constraints. For instance, although releasing in all patches is often optimal, targeting a few key patches may reduce operational costs, requiring a balance between production capacity, time to elimination, and field limitations.

Finally, as our model and results are generic, this approach could be applied to other SIT-related problems involving different pests or disease vectors, such as mosquitoes, provided that appropriate parametrisation is used.

Some perspectives for future work include the development of a sex- and stage-structured model for *n* patches, in order to incorporate control methods that target specific sexes and/or developmental stages. For instance, the Male Annihilation Technique is an attract-and-kill strategy that targets adult males, using attractants such as ME (Singh and Bajaj 2022). As another example, entomopathogenic fungi can be used as biological control agents. These fungi mainly affect pupal mortality, but also reduce adult survival (Li et al. 2024; Dumont 2025).

## A Theoretical Tools

In this section, we prove results that are useful for the analysis of model (6).

Consider the following system of ODEs:

A.34 $$\begin{aligned} \dot{x} = f(x), \end{aligned}$$

where $f: \mathcal {D} \rightarrow \mathbb {R}^n$ is differentiable and $\mathcal {D}$ is an open *p*-convex set of $\mathbb {R}^n$. Assume that all solutions are global.

### Theorem A.1

Assume system (A.34) is cooperative and irreducible on $\mathcal {D}$, $\mathcal {D}$ is positively invariant with respect to (A.34) and let $x^*$ be an equilibrium. Then, for any $x' \in \mathcal {D}$ such that $f(x') \in \mathbb {R}^n_+$, the following holds:

$$ x^* > x' \Rightarrow x^* \gg x'. $$

### Proof

Let $$x_0 > x'$$. Then, as a consequence of Theorem 1.1 in (Smith, 1995, 1995, Chapter 4), any solutions $$x(t,x_0)$$ and $x(t,x^{\prime })$ of system (A.34) with initial condition $$x_0$$ and $x^{\prime }$, respectively, satisfy

A.35 $$\begin{aligned} x(t,x_0) \gg x(t, x'), \quad t>0. \end{aligned}$$

Since $$f(x') \ge 0_n$$, then, by applying (Smith, 1995, Proposition 2.1, Chapter 3), the solution $x(t,x^{\prime })$ is non-decreasing, which implies $x\left(t,x^{\prime }\right)\geq x^{\prime }$ for all $$t \in \mathbb {R}_+$$. Therefore, by (A.35), it follows $$x(t,x_0) \gg x'$$ for all t>0. In particular, any equilibrium $$x^*$$ such that $$x^*> x'$$ must satisfy $$x^* \gg x'$$. □

Now consider the system

A.36 $$\begin{aligned} \dot{y} = g(y), \end{aligned}$$

where $g: \mathcal {D} \rightarrow \mathbb {R}^n$ is differentiable.

### Theorem A.2

Assume system (A.34) or system (A.36) is cooperative and irreducible on $\mathcal {D}$, $\mathcal {D}$ is positively invariant with respect to (A.34) and (A.36) and f(x)≤g(x) for every $x \in \mathcal {D}$. Then, for every $x_0 \in \mathcal {D}$, the following holds:

$$\begin{aligned} f(x_0) < g(x_0) \Rightarrow x(t,x_0) \ll y(t,x_0), \quad t>0, \end{aligned}$$

where $x(t,x_0)$ and $y(t,x_0)$ denote the respective solutions of (A.34) and (A.36) with initial condition $x_0$.

### Proof

Let $$x_0 \in \mathcal {D}$$. Since $$f(x_0) < g(x_0)$$, we have by continuity that $$x(t, x_0) < y(t,x_0)$$ on an interval (0,δ) where δ>0. Fix t>0. We can write $t=\left(t-t^{\prime }\right)+t^{\prime }$, where $$t' \in (0,\min \{t,\delta \})$$. Since $$x(t', x_0) < y( t',x_0)$$, we obtain by (Smith, 1995, Theorem 1.1, Chapter 4) that:

A.37 $$\begin{aligned} x\big (t - t', x(t',x_0)\big ) \ll x\big (t - t',y( t',x_0)\big ). \end{aligned}$$

Since system (A.34) or (A.36) is cooperative on $\mathcal {D}$ with f≤g, we also have by (Coppel, 1965, Theorem 10) that

A.38 $$\begin{aligned} x\big (t - t',y(t',x_0)\big )\le y\big (t - t',y( t',x_0)\big ). \end{aligned}$$

Combining (A.37) and (A.38) implies

$$ x\big (t - t', x( t',x_0)\big ) \ll y\big (t - t',y( t',x_0)\big ). $$

On the other hand, since the systems (A.34) and (A.36) are autonomous, we have $x\big (t - t', x( t',x_0)\big ) = x(t - t' + t',x_0) = x(t,x_0)$ and similarly, $y\big (t - t', y( t',x_0)\big ) = y(t,x_0)$. This yields

$$ x(t,x_0) \ll y(t,x_0). $$

□

## B One-Patch Model (Theorem 3.2)

### B.1 Proof of Theorem 3.2

#### Proof

Assume a>0. First, the derivative of the right-hand side of (1) when x=0 is equal to $-\mu _1 - \rho < 0$, which implies that 0 is LAS. Moreover, the positive equilibria of (1) are exactly the values x>0 such that

$$ b\frac{x}{x + a} - \mu _1 - \rho - \mu _2 x = 0, $$

that is, the positive roots of the polynomial equation

B.39 $$\begin{aligned} \frac{x^2}{Q} + \left( \frac{a}{Q}+ 1 - \mathcal {N} \right) x + a = 0. \end{aligned}$$

Since Qa>0, the roots of this equation, when real, have the same sign. Moreover, the sum of the roots is equal to $Q(\mathcal {N}-1)-a$, which implies that the roots, when real, are both negative when $\mathcal {N} \le 1$. In this case, system (1) admits no positive equilibrium. Since the polynomial in (B.39) is positive on $\mathbb {R}_+$, the origin of (1) is then GAS on $\mathbb {R}_+$.

Now assume $\mathcal {N} > 1$. The discriminant of Eq. B.39 is

$$\begin{aligned} \Delta&:=\left( \frac{a}{Q}+ 1 - \mathcal {N} \right) ^2 - \frac{4a}{Q} \\&= \frac{1}{Q^2} \left( a - a^\text {crit} \right) \left( a - a_+ \right) , \end{aligned}$$

where

$$ a_+:= Q\left( \sqrt{\mathcal {N}} + 1\right) ^2 > a^\text {crit}. $$

∙ When $0< a < a^\text {crit}$ or $a > a_+$, we have Δ>0, so the polynomial equation (B.39) admits two real roots, given by

$$\begin{aligned} x_\pm&= \frac{Q}{2} \left( \mathcal {N}-1 - \frac{a}{Q} \pm \sqrt{\left( \mathcal {N} - 1- \frac{a}{Q} \right) ^2 - \frac{4a}{Q}} \right) \\&= \frac{Q}{2} \left( \mathcal {N} - 1 - \frac{a}{Q}\right) \left( 1 \pm \sqrt{1-\frac{4a}{Q\left( \mathcal {N}-1 - \frac{a}{Q}\right) ^2}} \right) . \end{aligned}$$

If $$a > a_+$$, then $$\mathcal {N} - 1 - \frac{a}{Q} < 0$$, which implies that the two roots are negative, and there is no positive equilibrium. The origin is then GAS on $$\mathbb {R}_+$$. If, on the other hand, $$0< a < a^{\text {crit}}$$, then $$\mathcal {N} - 1 - \frac{a}{Q} > 0$$, and the two roots are positive. Since the polynomial in (B.39) is negative between the roots and positive outside, it follows that $$x_+$$ is LAS, $$x_-$$ is unstable, and the basins of attraction of 0 and $$x_+$$ are $$[0,x_-)$$ and $$(x_-,+\infty )$$, respectively.

∙ When $a = a^{\text {crit}}$, then Δ=0. Therefore, the equation (B.39) admits a unique root, given by

$$ x^*:= Q \left( \sqrt{\mathcal {N}}-1 \right) . $$

Since $$\mathcal {N} > 1$$, this root is positive, and thus the polynomial in (B.39) is non-negative. It follows that the basins of attraction of 0 and $$x^*$$ in $$\mathbb {R}_+$$ are $$[0, x^*)$$ and $$[x^*,+\infty )$$, respectively.

∙ When $a = a_+$, then Δ=0, and once again, (B.39) admits a unique root, equal to $-Q \left( \sqrt{\mathcal {N}}+1\right)$. Since this root is negative, there is no positive equilibrium.

∙ When $a \in (a^\text {crit}, a_+)$, then Δ<0, which implies that (B.39) admits no real root, and the system (1) admits no positive equilibrium. □

### B.2 Proof of Theorem 3.3

If (5) holds, then by (4), g(x)<0 for all $x \in \mathbb {R}_+$. Therefore, for any $x_0 \in \mathbb {R}_+$, the solution $x(t,x_0)$ of (1) with initial condition $x_0$ is strictly decreasing. This implies that the origin of (1) is GAS on $\mathbb {R}_+$.

Now assume $\mathcal {N}> 1$. To prove that the condition (5) is also necessary for the origin of (1) to be GAS on $\mathbb {R}_+$, we simply show that

B.40 $$\begin{aligned} a > a^\text {crit} \Rightarrow - \mu _1 - \rho +\left( \left( \sqrt{b} - \sqrt{a \mu _2} \right) ^+\right) ^2 < 0, \end{aligned}$$

so that we can apply $\textit{2.(a)}$ in Theorem 3.2.

Assume $$a> a^\text {crit}$$.

- If $a \ge \mathcal {N} Q$, then $- \mu _1-\rho + \left( \left( \sqrt{b} - \sqrt{a \mu _2} \right) ^+\right) ^2 = - \mu _1 - \rho < 0$, and thus (B.40) is satisfied.
- If $0 \le a < \mathcal {N}Q$, then $- \mu _1-\rho + \left( \left( \sqrt{b} - \sqrt{a \mu _2} \right) ^+\right) ^2 < 0$ is equivalent to
  B.41 $$\begin{aligned} -\mu _1 - \rho + b + a\mu _2 - 2\sqrt{a \mu _2 b } < 0. \end{aligned}$$
  Let $X:= \sqrt{a \mu _2}$. The inequality (B.41) is then equivalent to
  $$\begin{aligned} X^2-2\sqrt{b}X + b-\mu _1- \rho < 0. \end{aligned}$$
  The discriminant of this polynomial is equal to $4(\mu _1+\rho )$, so it admits two roots
  $$X_\pm := \sqrt{b} \pm \sqrt{\mu _1+\rho },$$
  which are positive since $\mathcal {N} >1$. We deduce that (B.41) is equivalent to
  $$\begin{aligned}&\sqrt{a} > \frac{\sqrt{b} - \sqrt{\mu _1+ \rho }}{\sqrt{\mu _2}} = \sqrt{Q}(\sqrt{\mathcal {N}}-1) \qquad \text {and}\\&\sqrt{a} < \frac{\sqrt{b} + \sqrt{\mu _1+ \rho }}{\sqrt{\mu _2}}= \sqrt{Q}(\sqrt{\mathcal {N}}+1), \end{aligned}$$
  and thus
  $$ a > a^\text {crit} \qquad \text {and} \qquad a < Q(\sqrt{\mathcal {N}}+1)^2. $$
  The second inequality is satisfied since $a < \mathcal {N}Q$, and thus we deduce that (B.40) holds.

## C Basin of Attraction (Theorem 3.5)

Since *D* is irreducible, the matrix $J_a$ is also irreducible. Then, by the extension of the Perron-Frobenius theorem, there exists a vector $c \gg 0_n$, such that

$$ c^{\textsf {T}} J_a = -\lambda c^{\textsf {T}}, \quad \text {with } \lambda := -s(J_a)> 0. $$

Up to multiplying *c* by $\frac{1}{\min _i c_i}$, we assume that $\min _i c_i = 1$, as done in the statement.

For any $x\in \mathbb {R}^n_+$, now consider the Lyapunov function candidate

$$ V(x):= c^{\textsf {T}} x. $$

The system (6) rewrites

$$ \dot{x} = J_ax + \textrm{diag}\biggl (\Big (b_i\left[ p(x_i,a_i) - p_{a_i}(0)\right] - \mu _{2,i} x_i\Big )x_i\biggr ). $$

Therefore, the derivative of *V* along a trajectory of (6) satisfies

$$\begin{aligned} \dot{V}(x)&= - \lambda c^{\textsf {T}} x + \sum _{i = 1}^{n} c_i x_i \big (b_i\left[ p(x_i,a_i) - p_{a_i}(0)\right] - \mu _{2,i} x_i\big ) \\&\le - \lambda c^{\textsf {T}} x + \sum _{i = 1}^{n} c_i x_i \max _{1 \le k \le n} \left\{ b_k\left[ p_{a_k}(x_k) - p_{a_k}(0)\right] - \mu _{2,k} x_k\right\} \\&= c^{\textsf {T}} x \left( -\lambda + \max _{1 \le k \le n} \left\{ b_k\left[ p_{a_k}(x_k) - p_{a_k}(0)\right] - \mu _{2,k} x_k\right\} \right) . \end{aligned}$$

Since for all k=1,…,n, $x_k \le \frac{c^{\textsf {T}} x}{\min _{i}c_i} = c^{\textsf {T}} x$ because $\min _{i}c_i = 1$, we have

$$\begin{aligned} \dot{V}(x)&\le c^{\textsf {T}} x \left( -\lambda + \max _{1 \le k \le n} ~ \max _{0 \le z \le c^{\textsf {T}} x} \{ b_k\left[ p_{a_k}(z) - p_{a_k}(0)\right] - \mu _{2,k} z \} \right) . \end{aligned}$$

∙ If $a_k = 0$, then $b_k\left[ p_{a_k}(z) - p_{a_k}(0)\right] - \mu _{2,k} z = -\mu _{2,k}z \le 0$. Thus, the maximum of the function $z \mapsto b_k\left[ p_{a_k}(z) - p_{a_k}(0)\right] - \mu _{2,k} z$ is attained at z=0 and is equal to 0.

∙ If $0< a_k < \frac{b_k}{\mu _{2,k}}$, then $b_k\left[ p_{a_k}(z) - p_{a_k}(0)\right] - \mu _{2,k} z = \frac{z}{z+a_k} - \mu _{2,k} z$. This function is increasing on $\left[ 0, -a_k + \sqrt{\frac{a_k b_k}{\mu _{2,k}}} \right]$ and decreasing on $\left[ -a_k + \sqrt{\frac{a_k b_k}{\mu _{2,k}}}, +\infty \right) ,$ with its maximal value, attained at $z^*_k = -a_k + \sqrt{\frac{a_k b_k}{\mu _{2,k}}}$, equal to $\left( \sqrt{b_k} - \sqrt{a_k \mu _{2,k}}\right) ^2$.

∙ If $a_k \ge \frac{b_k}{\mu _{2,k}}$, then the maximum is attained at z=0 and is equal to 0.

Therefore,

$$\begin{aligned} \max _{1 \le k \le n} ~ \max _{0 \le z \le c^{\textsf {T}} x} \{ b_k\left[ p_{a_k}(z) - p_{a_k}(0)\right] - \mu _{2,k} z \}&= \max _{\begin{array}{c} 1 \le k \le n \\ a_k> 0 \end{array}} ~ \max _{0 \le z \le c^{\textsf {T}} x} ~b_k\frac{z}{z+a_k} - \mu _{2,k} z\\ &= \max _{\begin{array}{c} 1 \le k \le n \\ a_k > 0 \end{array}} ~\phi _k( c^\top x). \end{aligned}$$

Thus, this implies that

$$ \dot{V}(x) \le c^{\textsf {T}} x \left( -\lambda + \Psi ( c^{\textsf {T}} x) \right) . $$

When $\Psi ( c^{\textsf {T}} x) < \lambda = -s(J_a)$, we have $\dot{V}(x) \le 0$ for all $x \in \mathbb {R}^n_+$. Then, since $c \gg 0_n$, $\dot{V}(x) = 0$ if and only if $x = 0_n$. Therefore, *V*(*x*) is a strict Lyapunov function on the set $\{ x \in \mathbb {R}^n_+: \Psi ( c^{\textsf {T}} x) < - s(J_a)\}$. This shows that the origin of (6) is GAS on this set, which proves that it is included in the basin of attraction $\mathcal {B}_a$ of the origin of (6). Moreover, since $\Psi (0) = 0 < -s(J_a)$, this set contains the origin. This completes the proof of Theorem 3.5.

## D Maximal Equilibrium (Theorem 3.6)

To prove that system (6) admits a maximal equilibrium $x_+(a)$, we first show that all the solutions of (6) are uniformly bounded. We compute the sum of the $\dot{x}_i$:

$$ \sum \limits _{\begin{array}{c} i=1 \end{array}}^{n}{\dot{x}_i} = \sum \limits _{\begin{array}{c} i=1 \end{array}}^{n}{ x_i \big (p(x_i,a_i) b_i - \mu _{1,i} - \rho _i- \mu _{2,i} x_i\big )} \le \sum \limits _{\begin{array}{c} i=1 \end{array}}^{n}{ x_i \left( b_i - \mu _{1,i} - \rho _i- \mu _{2,i} x_i\right) }. $$

Therefore, $$\sum \limits _{\begin{array}{c} i=1 \end{array}}^{n}{\dot{x}_i} < 0$$ for sufficiently large $x_i$, i=1,…,n, which implies that the solutions of (6) are uniformly bounded.

We can now prove the existence of a maximal equilibrium. Since the matrix $D - \textrm{diag}(\mu _{1,i}+\rho _i)$ is Metzler and Hurwitz, the extension of the Perron-Frobenius theorem implies the existence of a vector $v > 0_n$ such that

$$\begin{aligned} \big (D - \textrm{diag}(\mu _{1,i}+\rho _i)\big ) v < 0_n. \end{aligned}$$

Since the solutions of (6) are uniformly bounded, there exists λ>0 such that any equilibrium $x^*_a$ satisfies

D.42 $$\begin{aligned} x^*_a \le \lambda v. \end{aligned}$$

Moreover, as the right-hand side $f_a$ of system (6) satisfies $f_a \le f_0$ for any $a \in \mathbb {R}^n_+$, one can take λ>0 large enough such that

D.43 $$\begin{aligned} f_a(\lambda v) \le f_0(\lambda v) = \lambda \textrm{diag}(b_i - \mu _{2,i}\lambda v_i) v+ \lambda \big (D - \textrm{diag}(\mu _{1,i}+\rho _i)\big )v < 0_n. \end{aligned}$$

Since the solution of (6) with initial condition λv is decreasing, it converges towards a non-negative equilibrium $x_+(a)$. Let $x^*_a$ be another non-negative equilibrium. By (D.42), it satisfies $x^*_a \le \lambda v$. By cooperativeness, this implies that, at any time, the solution initiated in λv remains above $x^*_a$, and hence $x^*_a \le x_+(a)$. Moreover, by (D.43) and by (Anguelov et al., 2012, Theorem 6), $x_+(a)$ is GAS on the set $\{x \ge x_+(a) \}$.

Last, when *D* is irreducible, (10) is a direct consequence of Theorem A.1.

## E Monotonicity Properties (Theorems 3.9 and 3.11)

### E.1 Proof of Theorem 3.9

Let us prove that (13) holds. The right-hand side of (6) satisfies $$f_{a'} \le f_a$$ for all $$0_n \le a < a'$$. Then, for any point $$x_0 \in \mathbb {R}^n_+$$, the respective solutions $$x(t,x_0;a)$$ and $$x(t,x_0;a')$$ of model (6) with initial condition $$x_0$$ and corresponding to parameters *a* and $a^{\prime }$ satisfy

E.44 $$\begin{aligned} x(t,x_0;a') \le x(t,x_0;a), \quad t\ge 0. \end{aligned}$$

Assume $x_{0,i} \ge \max \{{x_+(a)}_i, {x_+(a')}_i\}$ for all i=1,…,n. Then by Theorem 3.6, it follows

$$ \lim _{t \rightarrow + \infty } x(t,x_0;a') = x_+(a'), \quad \lim _{t \rightarrow + \infty } x(t,x_0;a) = x_+(a). $$

By (E.44), this implies

E.45 $$\begin{aligned} x_+(a') \le x_+(a), \end{aligned}$$

so (13) holds.

Now assume *D* is irreducible. We prove that when $x_+(a) > 0_n$, the inequality ≤ in (13) is replaced by ≪.

If $$x_+(a) > 0_n$$, then, as the origin is always an equilibrium of (6), it follows by (10) in Theorem 3.6 that $$x_+(a) \gg 0_n$$. As a consequence, since $$f_{a'}(x) < f_a(x)$$ for every $$x \gg 0_n$$ and $a^{\prime }>a$, we have by Theorem A.2 that

$$ x\big (t,x_+(a);a'\big ) \ll x\big (t,x_+(a);a\big ), \quad t>0. $$

By (E.45) and by cooperativeness of the system, one has $x(t,x_+(a');a') \le x(t,x_+(a);a')$. Therefore,

$$ x\big (t,x_+(a');a'\big ) \ll x\big (t,x_+(a);a\big ), \quad t>0. $$

By definition of an equilibrium, we have $x\big (t,x_+(a');a'\big ) = x_+(a')$ and $x\big (t,x_+(a);a\big ) = x_+(a)$, and thus

$$ x_+(a') \ll x_+(a). $$

Last, we show that the basin of attraction $$\mathcal {B}_a$$ of $$0_n$$ is non-decreasing with *a*. Let $a^{\prime }\geq a$, and let $$x_0 \in \mathcal {B}_a \subset \mathbb {R}^n_+$$. By definition of $$\mathcal {B}_a$$, $$x(t,x_0, a) \rightarrow 0_n$$ as $$t \rightarrow + \infty $$. Since $a^{\prime }\geq a$, $$f_{a'}\le f_a$$. Therefore, the monotony of system (6) implies $$x(t,x_0; a') \le x(t,x_0; a)$$. By comparison and since it can easily be shown that $$\mathbb {R}^n_+$$ is positively invariant with respect to system (6), $$x(t,x_0; a') \rightarrow 0_n$$ as $$t \rightarrow + \infty $$, which proves that $$x_0 \in \mathcal {B}_{a'}$$. This completes the proof of Theorem 3.9.

### E.2 Proof of Theorem 3.11

To prove Theorem 3.11, we study the function $a\in \mathbb {R}^n_+ \mapsto s(A(a))$. One has

$$\begin{aligned} A(a) = \textrm{diag}\big (\varphi _i\left( a_i \right) \big )- \textrm{diag}(\mu _{1,i}+\rho _i) + D, \end{aligned}$$

where, for every i=1,…,n, the function $\varphi _i$ is defined by

$$\begin{aligned} \varphi _i(z)&:= \left( \left( \sqrt{b_i} - \sqrt{z \mu _{2,i}} \right) ^+\right) ^2. \end{aligned}$$

Since $\varphi _i$ is non-increasing,

$$ a < a' \Rightarrow A(a') \le A(a). $$

Extending (Berman and Plemmons, 1994, Corollary 1.5, Chapter 2) to Metzler matrices then implies the monotonicity statement (14).

Moreover, $\varphi _i$ is continuous on $\mathbb {R}_+$, which implies that the function a↦A(a) is continuous on $\mathbb {R}^n_+$. Its derivative on $\left( 0, \frac{b_i}{\mu _{2,i}}\right]$ is:

E.46 $$\begin{aligned} \varphi _i'(z) = \mu _{2,i} - \sqrt{\dfrac{b_i \mu _{2,i}}{z}}, \quad z \in \left( 0, \dfrac{b_i}{\mu _{2,i}}\right] . \end{aligned}$$

This derivative is continuous, showing that $\varphi _i$ is continuously differentiable on $\left( 0, \frac{b_i}{\mu _{2,i}}\right]$. Moreover, $\varphi _i'(\frac{b_i}{\mu _{2,i}}) = 0$ and $\varphi _i$ is constant on $\left( \frac{b_i}{\mu _{2,i}}, +\infty \right)$. Thus $\varphi _i$ is continuously differentiable on $\mathbb {R}^*_+$, implying that the function a↦A(a) is continuously differentiable on $\{a \gg 0_n\}$. Notice, however, that $\varphi _i$ is not differentiable at z=0, since $\varphi _i'(z) \rightarrow -\infty$ as $z \rightarrow 0^+$.

For z>0, the second derivative is:

$$ \varphi _i''(z) = \left\{ \begin{array}{ll} \dfrac{1}{2z} \sqrt{\dfrac{b_i \mu _{2,i}}{z}} & \text{ if } 0 < z \le \dfrac{b_i}{\mu _{2,i}} \\ 0 & \text{ if } z > \dfrac{b_i}{\mu _{2,i}}. \end{array} \right. $$

Thus, $\varphi _i$ is piecewise twice continuously differentiable on $\mathbb {R}^*_+$, but not twice continuously differentiable on $\mathbb {R}^*_+$ since $\varphi _i''(z) \ne 0$ when $z = \frac{b_i}{\mu _{2,i}}$. This implies that a↦A(a) is piecewise twice continuously differentiable on the set $\{a\gg 0_n\}$.

Finally, $\varphi _i''(z) = \frac{1}{2z} \sqrt{\dfrac{b_i \mu _{2,i}}{z}} > 0$ for any $z \in \left( 0,\frac{b_i}{\mu _{2,i}}\right]$. Since in addition, $\varphi _i'$ is constant on the interval $\left( \frac{b_i}{\mu _{2,i}}, + \infty \right)$, and $\varphi _i$ is differentiable on $\mathbb {R}^*_+$, it follows that $\varphi _i$ is convex on $\mathbb {R}^*_+$. We now prove that $\varphi _i$ is convex on the whole domain $\mathbb {R}_+$. For any $z,z' \in \mathbb {R}_+$, there exist sequences $z_n$ and $z'_n \in \left( \mathbb {R}^*_+\right) ^\mathbb {N}$ such that $z_n \rightarrow z$ and $z_n' \rightarrow z'$ as $n \rightarrow \infty$. By continuity and by convexity of $\varphi _i$ on $\mathbb {R}^*_+$, for all t∈[0,1],

$$\begin{aligned} \varphi _i\big (t z +(1-t)z'\big )&= \lim _{t \rightarrow +\infty } \varphi _i\big (t z_n +(1-t)z'_n\big ) \le \lim _{t \rightarrow \infty } t \varphi _i( z_n) + (1-t) \varphi _i(z'_n) \\&= t \varphi _i(z) + (1-t) \varphi _i(z'), \end{aligned}$$

which proves that $\varphi _i$ is convex on the whole domain $\mathbb {R}_+$.

The convexity of *s*(*A*(*a*)) then follows from (Kirkland and Neumann, 2012, Theorem 3.3.5). Now assume that *D* is irreducible. Then as a consequence of (Kirkland and Neumann, 2012, Theorem 3.2.1), the stability modulus of *A*(*a*) inherits the differentiability properties of *A*(*a*). If moreover, $a_i < \frac{b_i}{\mu _{2,i}}$ for all i=1,…,n, then, since the function $\varphi _i$ is strictly decreasing on $\left[ 0, \frac{b_i}{\mu _{2,i}}\right)$,

$$a<a^{\prime }\Rightarrow A\left(a^{\prime }\right)<A\left(a\right).$$

This implies by (Nussbaum, 1986, Lemma 1.2) extended to Metzler matrices that the inequality ≤ in (14) may be replaced by <.

## F Sterile Insect Equilibrium for Reducible Network (Theorem 4.2 and Lemma 4.4)

### F.1 Proof of Theorem 4.2

Let $\mathcal {G}$ be an SCC of $\Gamma _s$. By definition of the set $\mathcal {G}^-$ of its in-neighbouring SCCs, one has $d^s_{ji} = 0$ for every $j \in V_{\mathcal {G}^-}$ and $i \in V_{\mathcal {G}}$. Similarly, one has $d^s_{ij} = 0$ for every $j \in V_{\mathcal {G}^+}$ and $i \in V_{\mathcal {G}}$. It follows that, for any SCC $\mathcal {G}$ of $\Gamma _s$, the system (15) writes

$$\begin{aligned} \dot{x}_{s,i} &  = \Lambda _i - \left( \mu _{s,i} + \rho _{s,i} +\sum \limits _{\begin{array}{c} j \in V_{\mathcal {G}^+} \end{array}} {d^s_{ji}}\right) x_{s,i} \\ &  + \sum \limits _{\begin{array}{c} j \in V_{\mathcal {G}} \\ j \ne i \end{array}}{d^s_{ij} x_{s,j}} -\sum \limits _{\begin{array}{c} j \in V_{\mathcal {G}} \\ j \ne i \end{array}}{d^s_{ji} x_{s,i}} + \sum \limits _{\begin{array}{c} j \in V_{\mathcal {G}^-} \end{array}}{d^s_{ij} x_{s,j}}, ~~~~~i \in V_{\mathcal {G}}. \end{aligned}$$

To find the value of $x^*_s(\Lambda )|_{V_{\mathcal {G}}}$, we replace $x_{s,j}$ by $x^*_{s,j}(\Lambda )$ for every $j \in V_{\mathcal {G}^-}$. This results in the following system:

F.47 $$\begin{aligned} \dot{x}_{s,i} &  = \Lambda _i + \sum \limits _{\begin{array}{c} j \in V_{\mathcal {G}^-} \end{array}}{d^s_{ij} x^*_{s,j}(\Lambda )} \nonumber \\ &  - \left( \mu _{s,i} + \rho _{s,i} +\sum \limits _{\begin{array}{c} j \in V_{\mathcal {G}^+} \end{array}} {d^s_{ji}}\right) x_{s,i} + \sum \limits _{\begin{array}{c} j \in V_{\mathcal {G}} \\ j \ne i \end{array}}{d^s_{ij} x_{s,j}} -\sum \limits _{\begin{array}{c} j \in V_{\mathcal {G}} \\ j \ne i \end{array}}{d^s_{ji} x_{s,i}}, ~~~~~i \in V_{\mathcal {G}}. \end{aligned}$$

Therefore, the equilibrium of (F.47) satisfies (19). The matrix $D^s|_{V_{\mathcal {G}}}$ is irreducible by definition of an *SCC*, which implies that the matrix $J^s|_{V_{\mathcal {G}}}$ is also irreducible. Moreover, $J^s|_{V_{\mathcal {G}}}$ is a Metzler and Hurwitz matrix, so $- \left( J^s|_{V_{\mathcal {G}}}\right) ^{-1} \gg 0_{n_{V_{\mathcal {G}}} \times n_{V_{\mathcal {G}}}}$ by (Bullo et al., 2018, Theorem 10.3). We can then prove (20) in Theorem 4.2 by induction, following the acyclic ordering of the SCCs of the sterile insect network.

**Initialisation:** First, assume that $\mathcal {G}$ is of in-degree zero. Then $V_{\mathcal {G}_-} = \emptyset$, which implies by (19) that

$$\begin{aligned} x^*_s(\Lambda )|_{V_{\mathcal {G}}} = - \left( J^s|_{V_{\mathcal {G}}}\right) ^{-1} \Lambda |_{V_{\mathcal {G}}}. \end{aligned}$$

- Assume $V_{\mathcal {G}} \cap \mathcal {C}^s \ne \emptyset$. Then, $\Lambda |_{V_{\mathcal {G}}} > 0_{n_{V_{\mathcal {G}}}}$, which implies $x^*_s(\Lambda )|_{V_{\mathcal {G}}} \gg 0_{n_{V_{\mathcal {G}}}}$.
- Assume $V_{\mathcal {G}} \cap \mathcal {C}^s = \emptyset$. Then $\Lambda |_{V_{\mathcal {G}}} = 0_{n_{V_{\mathcal {G}}}}$, which implies $x^*_s(\Lambda )|_{V_{\mathcal {G}}} = 0_{n_{V_{\mathcal {G}}}}$.

This proves that (20) is true for any SCC of in-degree zero.

**Induction step:** Let $\mathcal {H}$ be an SCC of $\Gamma _s$ and assume (20) is true for any SCC $\mathcal {G}$ in $\mathcal {H}^{\text {up}}$.

- Assume $V_{\mathcal {H}} \cap \mathcal {C}^s \ne \emptyset$. Then, $\Lambda |_{V_{\mathcal {H}}} > 0_{n_{V_{\mathcal {H}}}}$. Therefore, by the formula in (19), $x^*_s(\Lambda )|_{V_{\mathcal {H}}} \gg 0_{n_{V_{\mathcal {H}}}}$.
- Assume $V_{\mathcal {H}^{\text {up}}} \cap \mathcal {C}^s \ne \emptyset$. Then, by definition of $\mathcal {H}^-$ and $\mathcal {H}^{\text {up}}$, there exists an SCC $\mathcal {G}$ in $\mathcal {H}^-$ such that $\{V_{\mathcal {G}} \cup V_{\mathcal {G}^{\text {up}}}\} \cap \mathcal {C}^s \ne \emptyset$. Since (20) is assumed true for $\mathcal {G}$, this implies that $x^*_s(\Lambda )|_{V_{\mathcal {G}}} \gg 0_{n_{V_{\mathcal {G}}}}$. Thus, since there exists $i \in V_{\mathcal {H}}$ and $j \in V_{\mathcal {G}}$ such that $d^s_{ij} > 0$, one has $$\Lambda |_{V_{\mathcal {H}}} + \left( \sum \limits _{\begin{array}{c} j \in V_{\mathcal {H}^-} \end{array}}{d^s_{ij} x^*_{s,j}(\Lambda )} \right) _{i \in V_{\mathcal {H}}} > 0_{n_{V_{\mathcal {H}}}}$$. This implies that $x^*_s(\Lambda )|_{V_{\mathcal {H}}} \gg 0_{n_{V_{\mathcal {H}}}}$.
- Assume $\{V_{\mathcal {H}} \cup V_{\mathcal {H}^{\text {up}}} \}\cap \mathcal {C}^s = \emptyset$. Then, $\Lambda |_{V_{\mathcal {H}}} = 0_{n_{V_{\mathcal {H}}}}$, and for any SCC $\mathcal {G}$ in $\mathcal {H}^-$, one has $\{V_{\mathcal {G}} \cup V_{\mathcal {G}^{\text {up}}}\} \cap \mathcal {C}^s = \emptyset$. Since (20) is assumed true for $\mathcal {G}$, this implies that $x^*_s(\Lambda )|_{V_{\mathcal {G}}} = 0_{n_{V_{\mathcal {G}}}}$. Therefore, $$\Lambda |_{V_{\mathcal {H}}} + \left( \sum \limits _{\begin{array}{c} j \in V_{\mathcal {H}^-} \end{array}}{d^s_{ij} x^*_{s,j}(\Lambda )} \right) _{i \in V_{\mathcal {H}}} = 0_{n_{V_{\mathcal {H}}}}$$, implying that $x^*_s(\Lambda )|_{V_{\mathcal {H}}} = 0_{n_{V_{\mathcal {H}}}}$.

By induction, this concludes the proof of Theorem 4.2.

### F.2 Proof of Lemma 4.4

By Theorem 4.2, for any SCC $\mathcal {G}$ of $\Gamma _s$, one has

$$ x^*_s(\Lambda )|_{V_{\mathcal {G}}} = - \left( J^s|_{V_{\mathcal {G}}}\right) ^{-1} \left( \Lambda |_{V_{\mathcal {G}}} + \left( \sum \limits _{\begin{array}{c} j \in V_{\mathcal {G}^-} \end{array}}{d^s_{ij} x^*_{s,j}(\Lambda )} \right) _{i \in V_{\mathcal {G}}} \right) , $$

with $- \left( J^s|_{V_{\mathcal {G}}}\right) ^{-1} \gg 0_{n_{V_{\mathcal {G}}} \times n_{V_{\mathcal {G}}}}$. Then, by reasoning by induction on the SCCs of $\Gamma _s$ as in the proof of Theorem 4.2, we obtain that, for all $k \in V_{\mathcal {G}}$:

- If $\Lambda _i \rightarrow + \infty$ for some $i \in V_{\mathcal {G}} \cup V_{\mathcal {G}^{\text {up}}}$, then, since $- \left( J^s|_{V_{\mathcal {G}}}\right) ^{-1} \gg 0_{n_{V_{\mathcal {G}}} \times n_{V_{\mathcal {G}}}}$, we have $x^*_{s,k}(\Lambda ) \rightarrow + \infty$.
- If $\Lambda _i = 0$ for all $i \in V_{\mathcal {G}} \cup V_{\mathcal {G}^{\text {up}}}$, then $x^*_{s,k}(\Lambda ) = 0$.
- If $\Lambda _i \rightarrow \overline{\Lambda }_i \in [0, + \infty )$ for all $i \in V_{\mathcal {G}} \cup V_{\mathcal {G}^{\text {up}}}$ with at least one $i \in V_{\mathcal {G}} \cup V_{\mathcal {G}^{\text {up}}}$ such that $\Lambda _i >0$, then since $- \left( J^s|_{V_{\mathcal {G}}}\right) ^{-1} \gg 0_{n_{V_{\mathcal {G}}} \times n_{V_{\mathcal {G}}}}$, it follows that $0< \lim _{\Lambda \rightarrow \overline{\Lambda }} x^*_{s,k}(\Lambda ) < +\infty$.

Using the definition of $\mathcal {C}^s, \mathcal {C}^s_I$ and $\mathcal {C}^s_B$ in Definition 4.1 along with the argument above allows to complete the proof of Lemma 4.4.

## G Gradient Evaluation (Lemma 6.3)

Let us compute the gradient of the function $h(\Lambda ) = s\big (A(a + \gamma x^*_s(\Lambda ))\big )$.

Since the matrix *D* is assumed irreducible, the matrix Q(Λ) admits zero as a simple eigenvalue for any $\Lambda \in \mathbb {R}^n_+$. Consequently, it admits a group inverse, denoted by $Q^\#(\Lambda )$.

For any $\Lambda \gg 0_n$, we have as a consequence of Theorem 4.2 that $x^*_s(\Lambda ) \gg 0_n$. The function *h* is therefore continuously differentiable with respect to $\Lambda \gg 0_n$ by Theorem 5.3, and the gradient $\nabla h(\Lambda )$ is then well-defined. Notice that we are only interested in strictly positive values of Λ, since admissible points for the interior-point algorithm must strictly satisfy the inequality constraints.

For any $\Lambda \gg 0_n$, we have by the chain rule that

G.48 $$\begin{aligned} \big (\nabla h(\Lambda )\big )_i = \sum \limits _{p = 1}^n \frac{\partial A_{pp} (a + \gamma x^*_s(\Lambda ))}{\partial \Lambda _i} \frac{\partial s}{\partial _{pp}}\big (A(a+\gamma x^*_s(\Lambda ))\big ), \qquad i = 1,\ldots ,n, \end{aligned}$$

where $\frac{\partial A_{pp} (a + \gamma x^*_s(\Lambda ))}{\partial \Lambda _i}$ is the partial derivative of the (*p*, *p*)-th entry of $A(a + \gamma x^*_s(\Lambda ))$ with respect to $\Lambda _i$, and $\frac{\partial s}{\partial _{pp}}\big (A(a+\gamma x^*_s(\Lambda ))\big )$ is the partial derivative of the stability modulus with respect to the (*p*, *p*)-th entry of $A(a + \gamma x^*_s(\Lambda ))$.

By (Deutsch and Neumann, 1984, Lemma 3.1), one has

G.49 $$\begin{aligned} \frac{\partial s}{\partial _{pp}}\big (A(a+\gamma x^*_s(\Lambda ))\big ) = \left( I- Q(\Lambda ) Q^\#(\Lambda ) \right) _{pp}, \qquad p = 1,\ldots , n. \end{aligned}$$

On the other hand, for any $\Lambda \gg 0_n$, straightforward computations yields

G.50 $$\begin{aligned} \frac{\partial A_{pp} (a + \gamma x^*_s(\Lambda ))}{\partial \Lambda _i} = \gamma \mu _{2,p} \left( J^s\right) ^{-1}_{pi} \left( \sqrt{\frac{b_p }{\big (a_p + \gamma x^*_{s,p}(\Lambda )\big ) \mu _{2,p}}} - 1\right) ^+. \end{aligned}$$

Incorporating (G.49) and (G.50) into (G.48) allows to recover (31) in Lemma 6.3.
